# Overexpression of miR-124 enhances the therapeutic benefit of TMZ treatment in the orthotopic GBM mice model by inhibition of DNA damage repair

**DOI:** 10.1038/s41419-025-07363-z

**Published:** 2025-01-26

**Authors:** Yuchen Wei, Peng Wang, Jianhui Zhao, Xin Fan, Jun Jiang, Xiuli Mu, Yuzhou Wang, Angang Yang, Rui Zhang, Shijie Hu, Zhangyan Guo

**Affiliations:** 1 State Key Laboratory of Holistic Integrative Management of Gastrointestinal Cancers, Department of Biochemistry and Molecular Biology, Fourth Military Medical University, Xi’an, Shaanxi Province China; 2 Department of Neurosurgery, The First Medical Center of Chinese PLA General Hospital, Beijing, China; 3 Department of Critical Care Medicine, Hainan Hospital of Chinese PLA General Hospital, Sanya City, Hainan Province China; 4 Department of Otolaryngology Head and Neck Surgery, Tangdu Hospital, Fourth Military Medical University, Xi’an, Shaanxi Province China; 5 Department of Health Service, Base of Health Service, Fourth Military Medical University, Xi’an, Shaanxi Province China; 6 State Key Laboratory of Holistic Integrative Management of Gastrointestinal Cancers, Department of Immunology, Fourth Military Medical University, Xi’an, Shaanxi Province China; 7 Department of Neurosurgery, Xijing Hospital, Fourth Military Medical University, Xi’an, Shaanxi Province China

**Keywords:** CNS cancer, Cancer therapy

## Abstract

Glioblastoma (GBM) is the most common malignant primary brain cancer with poor prognosis due to the resistant to current treatments, including the first-line drug temozolomide (TMZ). Accordingly, it is urgent to clarify the mechanism of chemotherapeutic resistance to improve the survival rate of patients. In the present study, by integrating comprehensive non-coding RNA-seq data from multiple cohorts of GBM patients, we identified that a series of miRNAs are frequently downregulated in GBM patients compared with the control samples. Among them, a high level of miR-124 is closely associated with a favorable survival rate in the clinical patients. In the phenotype experiment, we demonstrated that miR-124 overexpression increases responsiveness of GBM cells to TMZ-induced cell death, and vice versa. In the mechanistic study, we for the first time identified that RAD51, a key functional molecule in DNA damage repair, is a novel and bona fide target of miR-124 in GBM cells. Given that other miR-124-regulated mechanisms on TMZ sensitivity have been reported, we performed recue experiment to demonstrate that RAD51 is essential for miR-124-mediated sensitivity to TMZ in GBM cells. More importantly, our in vivo functional experiment showed that combinational utilization of miR-124 overexpression and TMZ presents a synergetic therapeutic benefit in the orthotopic GBM mice model. Taken together, we rationally explained a novel and important mechanism of the miR-124-mediated high sensitivity to TMZ-induced cell death in GBM and provided evidence to support that miR-124-RAD51 regulatory axis could be a promising candidate in the comprehensive treatment with TMZ in GBM.

## Introduction

Glioblastoma Multiforme (GBM) is the most lethal primary brain tumor in the central nervous system (CNS). It has only a low median survival with no more than 18 months after the initial diagnosis. The major means of GBM treatment is surgery followed by radiotherapy and chemotherapy [1]. Due to the blood-brain barrier (BBB), it’s hard to deliver the therapeutic agent to the CNS [2]. Hitherto, the lipophilic, alkylating agent temozolomide (TMZ) is still the only first-line chemical drug in the GBM treatment [3]. A large amount of clinical data has demonstrated that utilization of TMZ can extend patient survival [4]. It adds methyl groups to the genomic DNA on the O6, N7 position of guanine, and the N3 position of adenine. Among these DNA methylations, the O6-methylguanine induced DNA damage accounts for most TMZ-induced cell death [5, 6]. And this cytotoxic effect can be attenuated by O6-methylguanine-DNA methyltransferase (MGMT) [7]. In the clinic, hypermethylation of the MGMT promoter region has been used as the most accurate predictor for outcome and benefit from TMZ [8]. However, even for gliomas with methylated MGMT promoters, a large number of patients are still non-responding to TMZ treatment, indicating that a high MGMT level cannot cover all mechanism for the TMZ responsiveness [9, 10]. More ongoing explored mechanisms may help us to improve the effectiveness of TMZ in the clinic.

MicroRNAs (miRNAs) are a class of small noncoding RNAs (19~22 nt) binding to the 3′-untranslated region (3′-UTR) of target genes, which either leads to mRNA degradation or inhibits protein translation [11]. Over the past two decades, the concept of microRNA has been immersed in the field of cancer biology [12]. Classification of oncomiR and tumor suppressive miRNA helps us to further understand the underlying mechanism of miRNA in development and progression of cancers [13, 14]. The high abundance of tissue-specific miRNAs plays a critical role on maintenance of tissue homeostasis and regulation of pathophysiological functions [15–18]. Previous studies reported that miR-124 is a specific and abundant microRNA in the central neuron system (CNS) and governs neural differentiation and lineage selection [19, 20]. It has been demonstrated that brain-enriched miR-124 inhibits stemness and invasiveness in brain tumor stem cells by inducing cell differentiation [21]. More importantly, a recent study reported that using a gain-of-function library screening strategy, miR-124 is identified as a potential tumor-suppressive miRNA for inhibiting malignant progression of GBM in vivo [22]. However, whether it can be used as a predictor of cancer progression or therapeutic responsiveness is unclear. Here, we wondered to clarify its functional link with chemotherapy responsiveness in GBM.

In the present study, by analyzing multiple cohorts derived database, we found that a series of miRNAs, including miR-124 are the potential biomarker for prognosis in GBM patients. Both of loss and gain of functional experiments showed that miR-124 overexpression enhances TMZ sensitivity by inhibition of DNA damage repair in GBM cells. Mechanistically, RAD51 is a bona fide target post-transcriptionally silenced by miR-124 in GBM cells. Importantly, the rescue experiment identified that RAD51 is essential for miR-124-mediated regulation of GBM to TMZ treatment in vitro and in vivo. Finally, we demonstrated that overexpression of miR-124 enhances therapeutic benefit of TMZ treatment in the orthotopic GBM mice model. Our findings demonstrates that dysregulation of the miR-124-RAD51 axis contributes to chemotherapy responsiveness in GBM and further emphasizes the importance of combinational utilization of the functional miRNA and TMZ in the treatment of GBM in clinic.

## Results

### The multiple cohort-based analysis identifies miR-124 as the potential indicator for prognosis in GBM patients

**Fig. 1:**
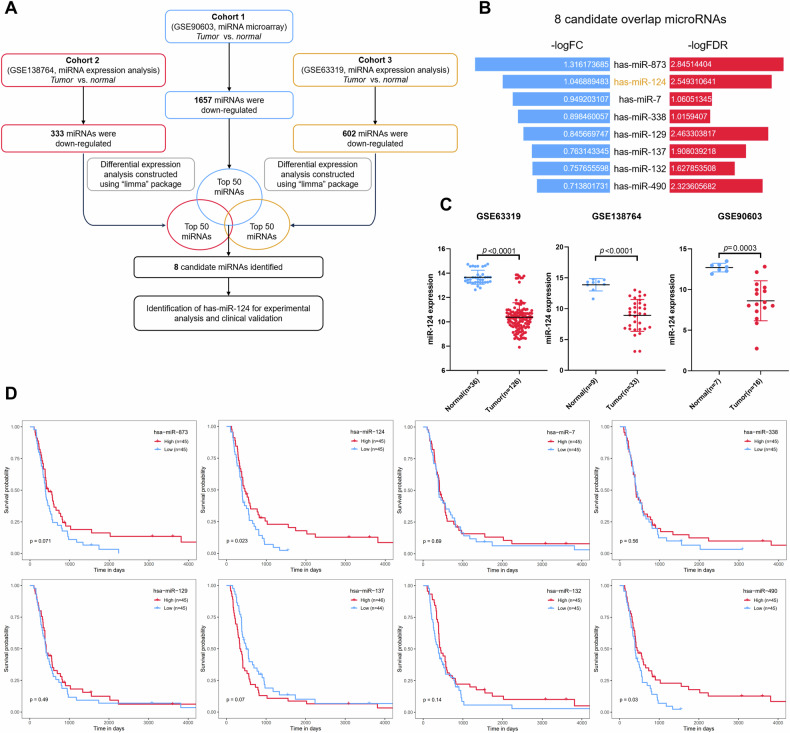
MiR-124 is identified as a potential tumor suppressor in GBM. **A** The workflow for the screening of downregulated microRNA in GBM. **B** Butterfly plot showing the median value of fold change (FC) and false discover rate (FDR) of screened 8 candidate microRNAs from intersection of three cohorts (GSE90603, GSE138764, GSE63319 datasets). **C** Scatter plot depicting the expression of miR-124 in normal versus tumor tissue samples from GEO database (GSE63319, GSE138764, GSE90603). **D** Kaplan–Meier survival analysis to investigate the associations of the expression levels of 8 candidate microRNAs with the overall survival of 90 grade IV patients from the CGGA database. The expression of 8 candidate microRNA was respectively divided into high and low groups according to their median values. *P* values were calculated using a log-rank test. The values of scatter plot were presented as means ± SD. *P* values were calculated using unpaired Student’s *t-*test.

Given previous studies used only one independent cohort to analyze the differential expression genes in GBM tissues in comparison with normal tissues, it may not rule out the biased sample selection and data analysis. We wondered to globally analyze the dysregulated miRNAs and deep explore their biological roles in advanced progression and therapeutic response in GBM patients. Accordingly, we employed the “limma” R package to construct differential expression genes analysis from three public cohorts. We found that 1657, 333 and 602 miRNAs are downregulated in these three GBM cohorts, respectively (Fig. 1A). Among them, we selected the top-50 ranking downregulated miRNAs from each cohort to analyze the overlapped subjects. And then, 8 miRNA candidates, including miR-873, miR-124, miR-7, miR-338, miR-129, miR-137, miR-132 and miR-490 were identified to all downregulated in three tested cohorts (Fig. 1B). To further estimate the prognostic value of these miRNA candidates in the clinic, we used the Kaplan–Meier plotter to analyze the contribution of these miRNAs for survival rate in the Chinese GBM Genome Atlas (CGGA) dataset. Interestingly, we found that miR-873, the most significant fold change and false discover rate miRNA, has no significance for survival analysis, whereas miR-124, ranking the second miRNA was significantly associated with poor prognosis in a low expression level (Fig. 1D). Accordingly, we focused on the function and regulatory mechanism of miR-124 in GBM. As shown in the tested datasets (GSE90603, GSE138746 and GSE63319), the miR-124 expression is in a significant low level in the tumors compared with normal tissues (Fig. 1C). Taken together, all these results indicated that miR-124 is a bona fide potential indicator for prognosis in GBM patients.

### MicroRNA-124 enhances TMZ-mediated cell death in GBM cells

**Fig. 2:**
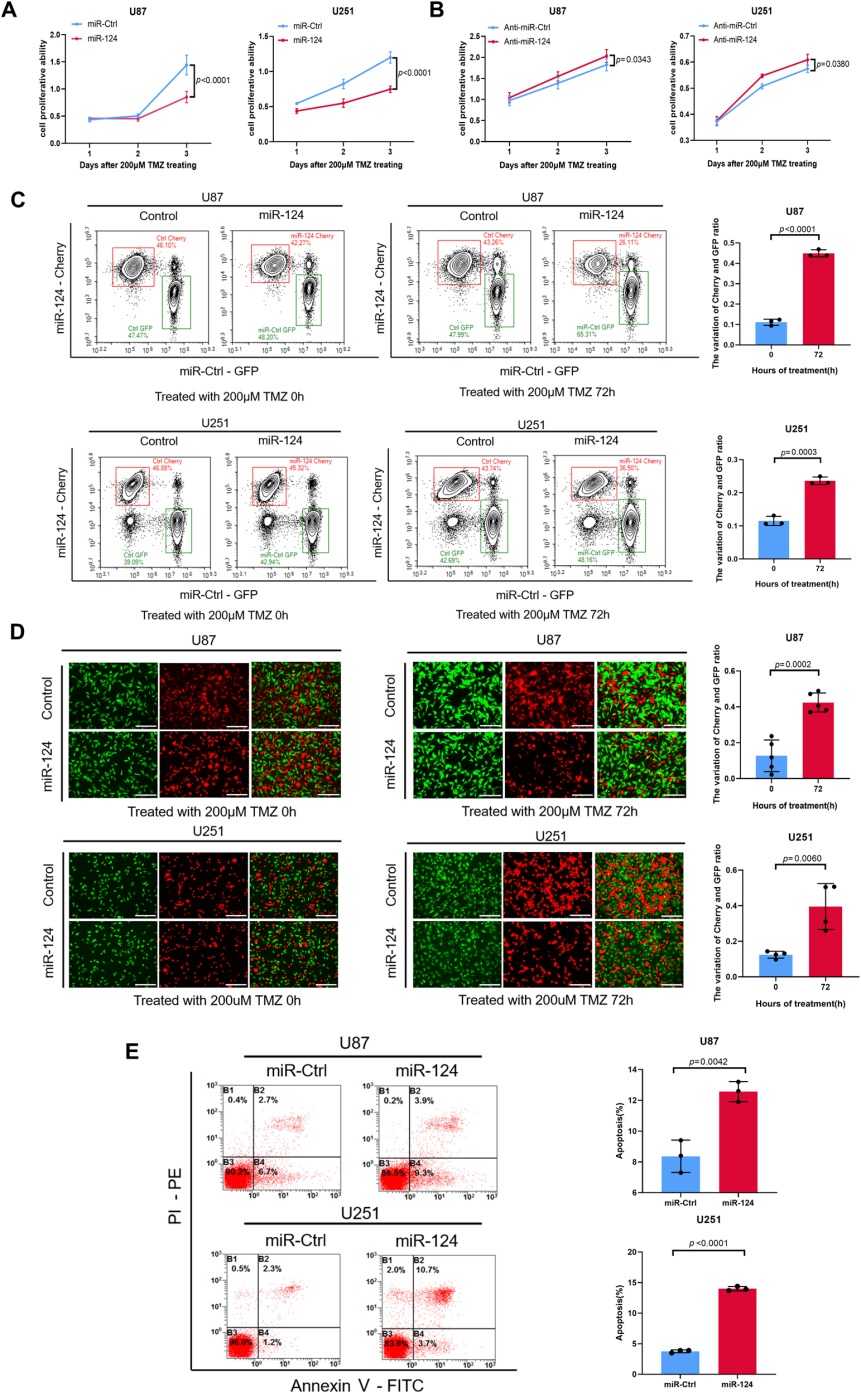
MiR-124 enhances the sensitivity of GBM cell lines to TMZ treatment. **A**, **B** CCK8 assay was used to assess the cell proliferative ability of different groups. U87 and U251 cells transfected with miR-Ctrl or miR-124 followed by TMZ treatment in (**A**), U87 and U251 cells transfected with anti-miR-Ctrl or anti-miR-124 followed by TMZ treatment in (**B**). Representative images of flow cytometry (**C**, left) and fluorescence imaging (**D**, left) and the variation ratio of Cherry/GFP cells (**C** and **D**, right) from co-culture cell competition assay in U87 and U251 cells exposed to 200 μM TMZ, with or without miR-124 overexpression. Scale bar, 300 μm. **E** PI/Annexin V-staining to detect the apoptosis of U87 and U251 cells exposed to TMZ (200 μM, 24 h) with or without miR-124 transfection (Representative images in left), Annexin V-stained (PI−/Annexin V+ and PI+/Annexin V+) cells were analyzed to measure the cell apoptosis rate (right). Above data are presented as means ± SD. All experiments were repeated at least 3 times. *P* values were calculated using unpaired Student’s *t-*test.

To explore the function of miR-124 in GBM cells, we increased exogenous expression of miR-124 by transfecting miR-124 mimics in U87 and U251 GBM cells lines. Although miR-124 has been documented as a tumor-suppressive miRNA in cancers, strikingly, we found that overexpression of miR-124 has no significant effect on cell proliferation in two tested GBM cell lines using the CCK-8 assay (Fig. S1A). Then, we asked whether miR-124 could have an influence on the activity of the first-line agent TMZ (Fig. 2A, B). GBM cells were treated with a range of concentrations of TMZ with or without the miR-124 mimic transfection, and the ability of TMZ to inhibit cell viability was assessed using the CCK-8 assay. As shown in the Fig. 2A, overexpression of miR-124 enhances TMZ-mediated inhibition on GBM cell proliferation. Vice versa, when we used miR-124 antagomiR to decrease the endogenous miR-124 in GBM cell lines, TMZ-mediated inhibition on GBM cell proliferation was significantly attenuated using CCK-8 assay (Fig. 2B). And the similar phenotypes were also observed with high dose TMZ treatment (Fig. S1B). These results indicated that miR-124 may sensitize the GBM cells to TMZ treatment rather than suppress tumor growth directly.

Furthermore, we established a co-culture cell competition assay to more accurately estimate the effect of miR-124 on responsiveness of GBM cells to the TMZ. Parental cells stably expressed green fluorescent protein EGFP or red fluorescent protein mCherry, and then fluorescent GBM cell lines were transfected with miR-124 or control mimics, respectively. Next, 1:1 mixed cells were treated with TMZ in the indicated dosage. As expected, the number of red cell was reduced and the variation of mCherry/GFP ratio was increased (Figs. 2C, D, S2A, B). The similar phenotypes were also observed with high-dose TMZ treatment (Fig. S1C, D). Overall, these data supported that miR-124 enhances TMZ sensitivity in GBM cells.

To further confirm the effect of miR-124 on the TMZ treatment in GBM cells, we utilized Annexin V staining to test its influence on TMZ-induced cell death. It was shown that the treatment of miR-124 plus TMZ significantly increases percentage of Annexin V positive cells compared with TMZ alone (Figs. 2E and S2E). Additionally, analysis of Western blot showed that overexpression of miR-124 increases cleavage of Caspase-3 in TMZ-treated GBM cells (Fig. S1E). Taken together, our data revealed that overexpression of miR-124 enhances TMZ-mediated cell death in GBM cells.

### MiR-124 sensitizes GBM cells to TMZ treatment by blocking DNA damage repair

**Fig. 3:**
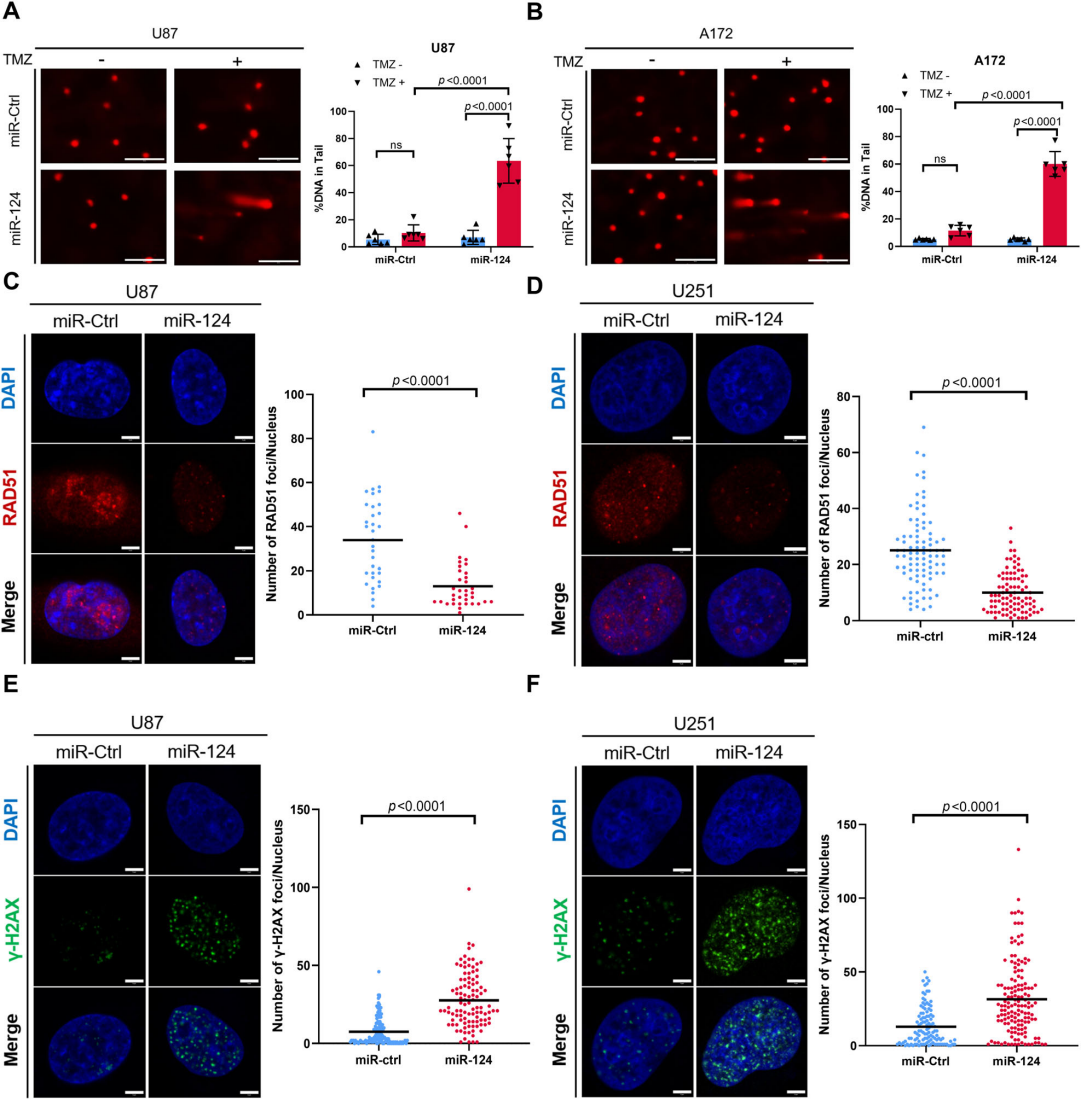
MiR-124 enhances the responses of TMZ in GBM cells by blocking DNA damage repair. **A**, **B** Representative images (left) and DNA tailing rate (right) from comet assay in U87 and A172 cells exposed to TMZ (500 μM, 4 h), with or without miR-124 overexpression. Scale bar, 200 μm (% DNA in tail: Tail DNA Intensity/Cell DNA Intensity × 100, miR-ctrl plus TMZ vs miR-124 plus TMZ, *P* < 0.0001). **C**, **D** Representative images (left) and quantification of RAD51 fluorescence foci (right) of immunofluorescence analysis in U87 and U251 cells exposed to TMZ (200 μM, 24 h) with or without miR-124 transfection. Scale bar, 5 μm. **E**, **F** Representative images (left) and quantification of γ-H2AX fluorescence foci (right) of immunofluorescence analysis in U87 and U251 cells exposed to TMZ (200 μM, 24 h) with or without miR-124 transfection. Scale bar, 5 μm. All experiments were repeated at least 3 times. Above data are presented as means ± SD. *P* values were calculated using unpaired Student’s *t-*test.

**Fig. 4:**
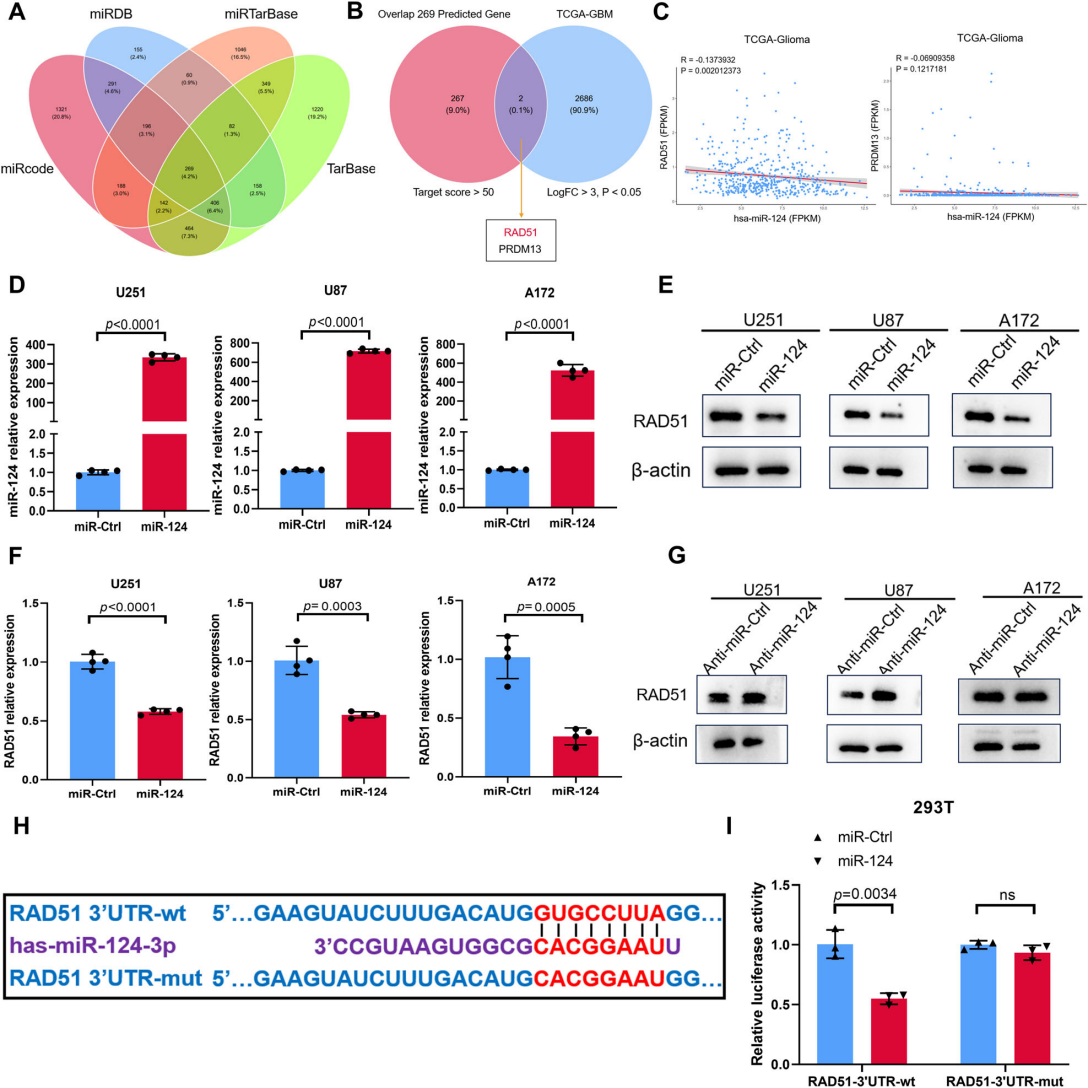
RAD51 expression is regulated by miR-124 in GBM cells. **A** Venn plot showing the overlap of four predicted miRNA-mRNA binding datasets (miRcode, miRTarBase, miRDB, TarBase). **B** Identification of potential target genes of miR-124. Only two genes (RAD51, PRDM13) were screened by overlapping the predicted genes (target score >50) in (**A**) with significant differential expression genes (LogFC >3, *P* < 0.05) in TCGA. **C** Correlation analysis between miR-124 and RAD51 or PRMD13 using TCGA database. A significant negative correlation was shown only for RAD51 and miR-124. *P* values were calculated using Pearson correlation test. **D** Quantitative real-time chain reaction (qRT-PCR) analysis of miR-124 expression in GBM cells transfected with miR-Ctrl or miR-124 mimics. **E**, **F** Western blot and qRT-PCR analysis of RAD51 expression in multiple GBM cell lines transfected with miR-Ctrl or miR-124 mimics. **G** Western blot analysis of RAD51 expression in GBM cells transfected with anti-miR-Ctrl or anti-miR-124. **H** Predication of one binding site of miR-124 on RAD51 3′-UTR by TargetScan. **I** Dual-luciferase reporter assay showing miR-124 directly targets the RAD51 3′-UTR. Firefly luciferase activity of the reporter was normalized to the internal Renilla luciferase activity. All experiments were repeated at least 3 times. All data are presented as means ± SD. *P* values were calculated using unpaired Student’s *t-*test.

Although it has been reported that miR-124 enhances TMZ-involved chemosensitivity by targeting Ras family members, Ras-driven intracellular signaling cannot rationally explain miR-124-involved regulation on TMZ responsiveness [23]. Given that the major mechanism of TMZ-induced cell death is DNA damage and known mechanisms of TMZ resistance are closely associated with the increasing activation of DNA damage repair [24], we wondered to known whether miR-124-mediated high responsiveness of GBM cells to TMZ is in a DNA damage repair dependent behavior. We measured the persistence of double-strand breaks after TMZ treatment as an indicator of unrepaired damaged DNA. Single-cell gel electrophoresis (alkaline comet assay) was carried out to measure DNA damage. GBM cells with ectopic overexpression of miR-124 had lower levels of RAD51 protein and statistically significantly higher residual DNA damage than control cells (Fig. 3A–D). Moreover, we examined the expression of γ-H2AX, a canonical biomarker for DNA damage by immunofluorescence staining. The results showed that a much higher level of γ-H2AX foci appears in GBM cells treated with overexpression of miR-124 plus TMZ in comparison with miR-ctrl plus TMZ (Fig. 4E, F). Taken together, these data indicated that miR-124 augments DNA damage in GBM cells with TMZ treatment. More importantly, among the reported mechanistic studies, there is no any report on how miR-124 affects TMZ responsiveness by modulating the DNA damage repair pathway.

### RAD51 is a novel target post-transcriptionally silenced by miR-124

To deep explore the underlying regulatory mechanism of miR-124 in GBM, four miRNA-mRNA binding prediction software (miRcode, miRDB, miRTarBase and TarBase) were used to predict the potential target genes regulated by miR-124. And 269 candidates were overlapped in all four-prediction software (Fig. 4A). We wondered to know whether the novel target(s) of miR-124 is upregulated in GBM tissues. Accordingly, in the TCGA GBM dataset, we set the parameters that fold change is more than 3 and *p-*value less than 0.05, and 2688 differential upregulated genes were screened. And then, we performed intersection analysis on the bioinformatic predicted target genes and the differential genes analyzed in the TCGA GBM database. RAD51 and PRDM13 were finally identified in the overlapped section (Fig. 4B). To further confirm the downstream gene, we used Pearson analysis to determine the relationship between miR-124 with RAD51 or PRDM13, respectively. Notably, the level of RAD51 expression in GBM was negatively correlated with the miR-124 expression, whereas the correlation between miR-124 and PRDM13 had no obvious significance (Fig. 4C).

To confirm the regulatory relationship between miR-124 and RAD51, we overexpressed miR-124 in a series of GBM cell lines and examined the expression of RAD51 using RT-qPCR and Western blot. As shown in Fig. 4D–F, when GBM cells were treated with miR-124 mimic, the mRNA and protein levels of RAD51 reduced significantly. Conversely, cancer cells transfected with miR-124 antagomiR expressed a much higher level of RAD51 than those transfected with the control mimics (Fig. 4G), indicating that RAD51 is negatively regulated by miR-124 in GBM cells. Next, we wondered to confirm the direct interaction between miR-124 and the RAD51 mRNA. The conserved targeting sequences for miR-124 were found in the 3′UTR of RAD51 mRNAs (Fig. 4H). This observation was further substantiated by the luciferase assay. 293 cells were transfected with the luciferase reporter plasmid containing the WT 3′UTR of RAD51 mRNA and miR-124 mimics, resulting in the inhibition of luciferase activity, whereas the luciferase activity of mutant 3′UTR was not suppressed by miR-124 (Fig. 4I). Collectively, these results suggested that RAD51 is a novel and bona fide target post-transcriptionally silenced by miR-124 in GBM cells.

### Inhibition of RAD51 sensitizes TMZ treatment in GBM cells by disrupting DNA damage repair

**Fig. 5:**
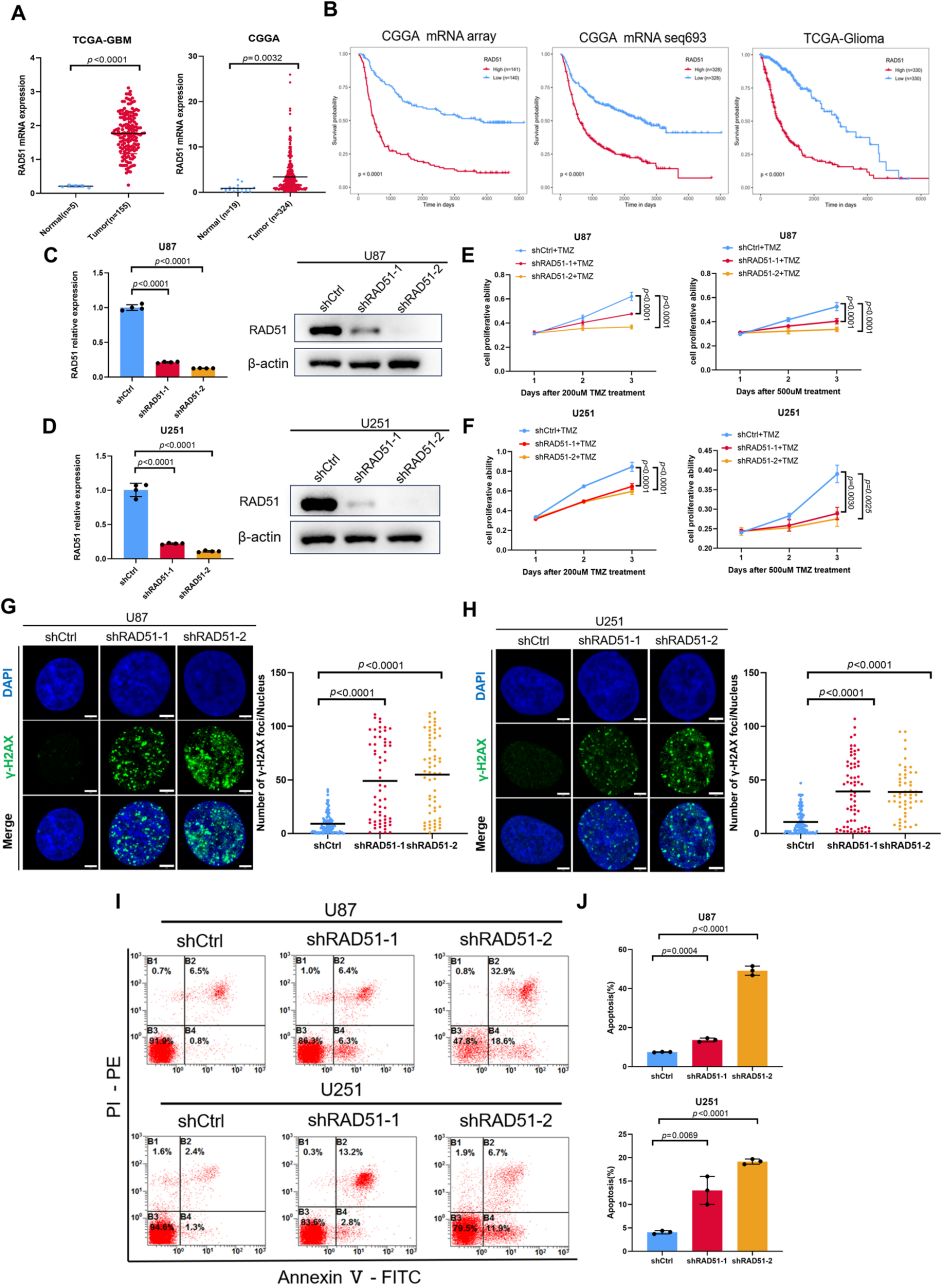
Knockdown of RAD51 enhances TMZ chemosensitivity in GBM cells. **A** Scatter plot showing the expression of RAD51 in normal versus tumor tissue samples from TCGA and CGGA databases. **B** Kaplan–Meier survival analysis investigate the associations of the expression levels of RAD51 with the overall survival from CGGA mRNA array (281 patients), CGGA mRNA-seq 693 (656 patients), TCGA (660 patients). The expression of RAD51 was divided into high and low groups according to its median value. *P* values were calculated using a log-rank test. **C**, **D** qRT-PCR (left) and western blot (right) validation of RAD51 expression in U87 and U251 cells with or without RAD51 knockdown. **E**, **F** CCK8 assay was used to assess cell viability of U87 and U251 cells with or without RAD51 knockdown treated with different dose of TMZ. **G**, **H** Representative images (left) and quantification of γ-H2AX fluorescence foci (right) of immunofluorescence analysis in U87 and U251 cells exposed to TMZ (200 μM, 24 h) with or without RAD51 knockdown. Scale bar, 5 μm. **I**, **J** Representative images (left) and apoptosis rate (right) of cell apoptosis assay in U87 and U251 cells exposed to TMZ (200 μM, 24 h) with or without RAD51 knockdown. All experiments were repeated at least 3 times. Above data are presented as means ± SD. *P* values were calculated using one-way ANOVA.

Given that we identified that RAD51 is a novel target of miR-124 in GBM cells, we wondered to identify the biological function of RAD51 in GBM. TCGA and CGGA datasets were applied to evaluate the expression of RAD51, and it was shown that the RAD51 level is significantly higher in GBM tissues compared with the normal ones (Fig. 5A). More importantly, it was found that a higher level of RAD51 is significantly associated with poor prognosis in GBM patients (Fig. 5B). To evaluate the biological contribution of RAD51 on TMZ responsiveness in GBM, we used two independent shRNAs to knock down RAD51 expression in U87 and U251 cell lines, respectively. The efficiency of RAD51 knockdown in GBM cell lines was validated by Western blot and RT-qPCR (Fig. 5C, D). Also, we performed qRT-PCR assay and identified that RAD51 knockdown has no significant effect on the miR-124 expression level in GBM cell lines (Fig. S2C, D). Using CCK-8 assay, we found that RAD51 knockdown obviously increased TMZ-mediated inhibition on GBM cell proliferation, emphasizing the importance of the regulatory role of RAD51 in TMZ-treated GBM cells (Fig. 5E, F). Furthermore, with the treatment of TMZ, we also used immunofluorescence staining of γ-H2AX to determine the influence of RAD51 on TMZ-induced DNA damage repair. As shown in Fig. 5G, H, RAD51 knockdown increased γ-H2AX foci in GBM cells. Finally, we determined the role of RAD51 inhibition on TMZ-induced GBM cell death. Accordingly, we used flow cytometry to evaluate the percentage of Annexin V positive cells in RAD51 knockdown GBM cells with the TMZ treatment (Fig. 5I, J). Our data revealed that RAD51 knockdown sensitizes TMZ-induced DNA damage and consequently results in more cell death in GBM cells.

### RAD51 is essential for miR-124 mediated regulation of GBM to TMZ treatment in vitro and in vivo

**Fig. 6:**
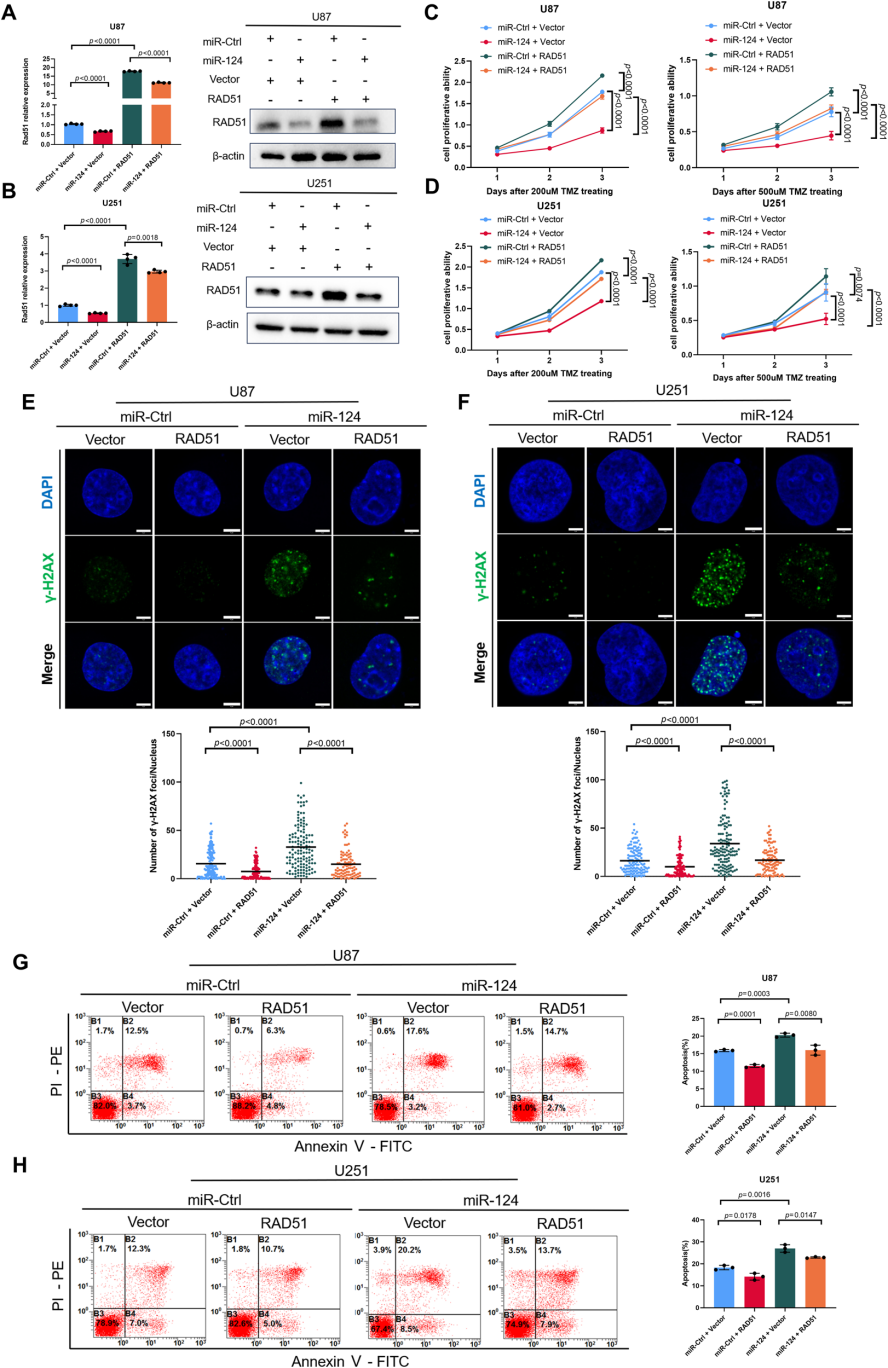
RAD51 is essential for miR-124 mediated sensitivity to TMZ treatment in GBM cells. **A**, **B** qRT-PCR (left) and western blot (right) validation of RAD51 expression in U87 and U251 cells transfected with miR-Ctrl or miR-124 together with empty vector or RAD51. **C**, **D** CCK8 assay was used to assess cell proliferative ability of U87 and U251 cells with or without miR-124 transfection together with or without RAD51 overexpression and treated with different dose TMZ. **E**, **F** Representative images (top) and quantification of γ-H2AX fluorescence foci (bottom) of immunofluorescence analysis in U87 and U251 cells exposed to TMZ (200 μM, 24 h), transfected with miR-Ctrl or miR-124 together with empty vector or RAD51. Scale bar, 5 μm. **G**, **H** Representative images (left) and apoptosis rate (right) of cell apoptosis assay in U87 and U251 cells exposed to TMZ (200 μM, 24 h), transfected with miR-Ctrl or miR-124 together with empty vector or RAD51. All experiments were repeated at least 3 times. Above data are presented as means ± SD. *P* values were calculated using unpaired Student’s *t-*test.

Given that we identified a miR-124-RAD51 regulatory axis in GBM cells, we wondered to know whether RAD51 is essential for mir-124 mediated regulation of GBM to TMZ treatment. We performed a rescue experiment to overexpress RAD51 in the miR-124 overexpressing GBM cells. The exogenous expression of RAD51 was validated by Western blot and RT-qPCR (Fig. 6A, B). Strikingly, using CCK-8 assay, we found that restoration of RAD51 significantly attenuates overexpression of miR-124 induced high responsiveness of GBM cells to TMZ treatment (Fig. 6C, D). Additionally, we used the immunofluorescence staining to test the effect of RAD51 on miR-124 sensitized DNA damage. The result showed that restoration of RAD51 inhibits γ-H2AX foci in miR-124 overexpressing GBM cells with the TMZ treatment (Fig. 6E, 6). Finally, we used FACS to test the influence of RAD51 on miR-124 promoted high responsiveness of GBM cells to TMZ. We found that rescue of RAD51 decreases percentage of Annexin V positive GBM cells treated with TMZ (Fig. 6G, H).

**Fig. 7:**
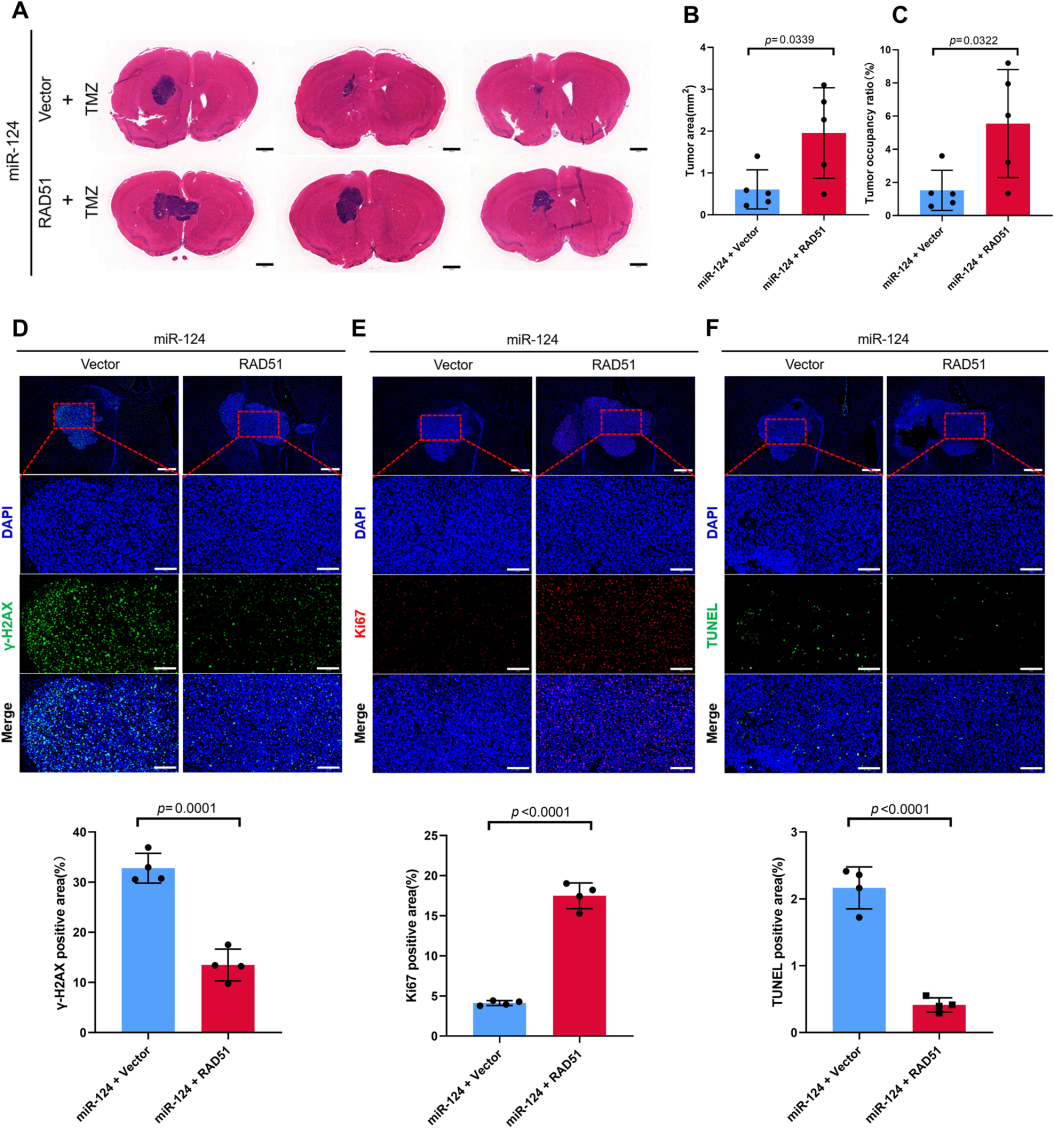
RAD51 is required for GBM resistant to TMZ treatment in vivo. **A** HE staining showing representative images (present 3 samples per group) of gross tumors (location near the lateral ventricle) from two rescue treatment groups in mouse orthotopic model. Scale bar, 1 mm. **B**, **C** Statistic of tumor size or tumor occupancy rate from two rescue treatment groups in (**C**) (*n* = 5 per group). **D**–**F** Representative images (top) and quantification of γ-H2AX, Ki67 and TUNEL positive immunofluorescence area (bottom) in the two rescue treatment groups. Scale bar in low-power microscope 500 μm, in high-power microscope 150 μm. All experiments were repeated at least 3 times. Above data are presented as means ± SD. *P* values were calculated using unpaired Student’s *t-*test.

To further verify the role of RAD51 in miR-124 involved regulation on TMZ treatment in vivo, we performed the rescue experiment by restoring RAD51 expression in vivo with TMZ treatment. Notably, HE staining showed that enforced expression of RAD51 in miR-124 overexpressing tumor tissues obviously attenuates TMZ-induced suppression on tumor growth, indicating the potential role of a miR-124-RAD51 axis in TMZ resistance in vivo (Fig. 7A–C). Moreover, the result of immunofluorescence staining of γ-H2AX, Ki67 and TUNEL showed that restoring RAD51 expression decreases DNA damage and cell apoptosis, and concurrently increases the tumor growth index (Fig. 7D–F). Together, these results demonstrated that RAD51 is essential for miR-124 mediated regulation of GBM to TMZ treatment in vitro and in vivo.

### Therapeutic benefit of the combinational utilization of mR-124 and TMZ in the orthotopic GBM mice model

**Fig. 8:**
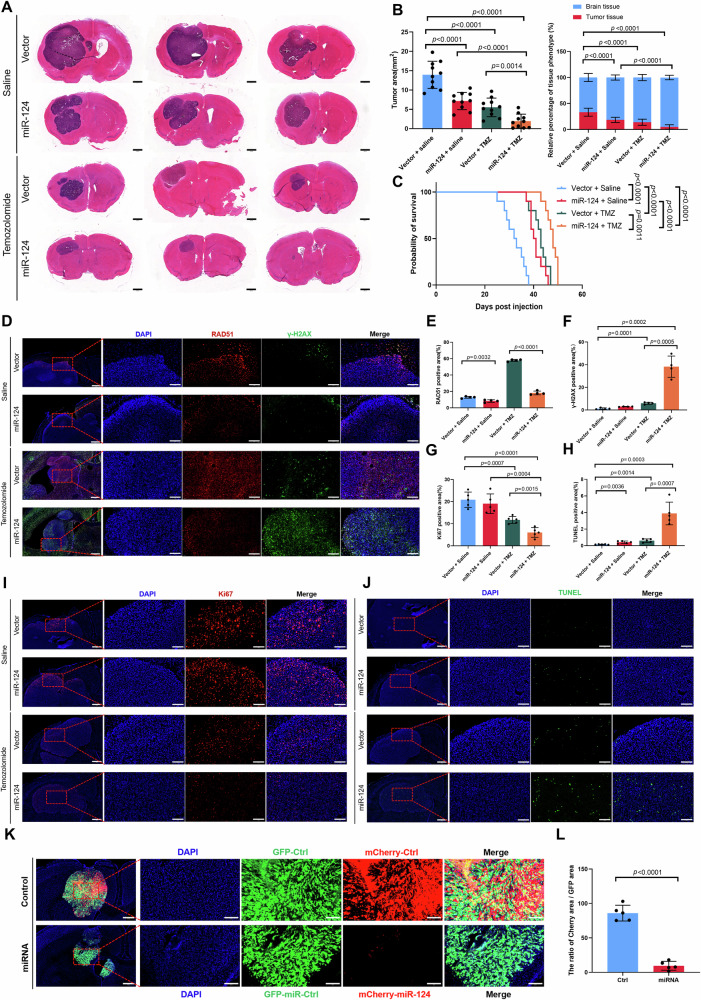
MiR-124 sensitizes TMZ therapy in GBM mouse model. **A** Hematoxylin Eosin (HE) staining showing representative images (present 3 samples per group) of gross tumors (location near the lateral ventricle) from different treatment groups in mouse orthotopic model. Scale bar, 1 mm. **B** Statistic of tumor size or tissue phenotype percentage from different treatment group in (**A**) (*n* = 10 per group). **C** Kaplan–Meier survival analysis to assess survival of mice in the four-treatment groups. *P* values were calculated using a log-rank test. Representative images (**D**) and quantification of RAD51 (**E**) and γ-H2AX (**F**) positive immunofluorescence area in the four-treatment group. Scale bar in low-power microscope 500 μm, in high-power microscope 150 μm. Representative images (**I**, **J**) and quantification of Ki67 (**G**) and TUNEL (**H**) positive immunofluorescence area in the four-treatment group by continuous frozen section. Scale bar in low-power microscope 500 μm, in high-power microscope 150 μm. Representative images (**K**) and quantification of the Cherry/GFP area rate (**L**) of coculture fluorescence competition assay in vivo. Scale bar in low-power microscope 500 μm, in high-power microscope 150 μm. All experiments were repeated at least 3 times. Above data are presented as means ± SD. *P* values were calculated using unpaired Student’s *t*-test.

To verify the effect of miR-124 in vivo, we applied stereotaxic apparatus to establish an intracranial orthotopic GBM mice model. U87 tumor-bearing mice were divided into four groups, including “saline plus Vector”, “saline plus miR-124”, “TMZ plus Vector” and “TMZ plus miR-124”. As shown in the HE staining, data of the tumor size and tumor occupancy rate showed that tumorigenicity is most significantly delayed in the TMZ plus miR-124 treated group (Fig. 8A, B). More importantly, Kaplan–Meier plotter analysis showed that the combinational utilization of miR-124 and TMZ potentially prolongs the survival time of tumor-bearing mice among four groups (Fig. 8C). Furthermore, data of the immunofluorescence staining showed that the expression of RAD51 obviously decreases in the miR-124 overexpressing xenografts. In addition, the result of immunofluorescence staining on γ-H2AX signaling showed that combinational treatment group leads to more DNA damage phenotype than TMZ or miR-124 treatment alone (Fig. 8D–F). Additionally, Ki67 and TUNEL staining in tumor tissue showed that the TMZ plus miR-124 treated group has a more effect on inhibition of cancer cell proliferation and induction of cancer cell death in vivo (Fig. 8G–J). Finally, we used a co-culture fluorescence competition assay in vivo to validate therapeutic benefit of the combinational utilization of mR-124 and TMZ in the orthotopic GBM model. The result showed that xenografts expressing miR-124 and mCherry fluorescent protein was almost eliminated, compared with the tumor tissues only expressing EGFP fluorescent protein (Figs. 8K, L, S2F). Together, these results demonstrated that miR-124 potently augments TMZ treatment efficiency in vivo.

## Discussion

In the present study, we interrogated multiple independent cohorts of clinically annotated genomics datasets (GEO) and showed that miR-124 expression is a top rank of frequently downregulated miRNA. Furthermore, we found that a high level of miR-124 in GBM tissues is closely associated with longer survival in GBM patients. Although a series of previous studies have reported the tumor suppressive roles of miR-124 in multiple cancers, including GBM [25–30], we for the first time showed the prognostic value of it in the clinic. More importantly, our study demonstrated that, except for the previous reported inhibition on GBM stemness and cancer cell invasion, the DNA damage repair is also blocked after overexpression of miR-124 in GBM cells treated with TMZ. In computational and experimental analyses, we identified that miR-124 directly targets RAD51 and sensitizes GBM cells to DNA damage and TMZ-induced cell death. In the preclinical study, we showed that overexpression of miR-124 enhances the effect of TMZ in an orthotopic GBM mouse model. The discovery of a miR-124-RAD51-DNA repair axis supports the strategy that combining miR-124 overexpression with DNA-damaging agents TMZ may substantially benefit GBM management (Fig. S4).

Although DNA-damaging agents are important chemotherapeutic interventions for cancer therapy, chemoresistance is still a major obstacle blocking the clinical benefit in GBM [24]. Homologous recombination is involved in tumor chemoresistance, and RAD51 is one of hub components of the homologous recombination-mediated double-strand DNA break repair machinery. It assembles onto single-stranded DNA as nucleoprotein filaments and catalyzes the exchange of homologous DNA sequences [31]. We showed that the RAD51 expression is frequently higher in tumor tissues as compared with normal ones and a high level of RAD51 is closely associated with an unfavorable prognosis in cancers, including malignant gliomas (Fig. S3). And accumulative evidence has demonstrated that RAD51 inhibition sensitizes tumor cells to DNA-damaging agents [32]. It was showed that a high level of RAD51 attribute to chemoresistance in ovarian cancer and soft tissue sarcoma [33–35]. And the expression pattern of RAD51 is inversely correlated with efficacy of radiotherapy or chemotherapy in glioma cells [36]. Currently, a series of mechanistic studies reveals the reasons of high levels of RAD51 in different types of cancers. It was reported that miR-506 sensitizes ovarian cancer cells to platinum treatment through directly targeting RAD51 [33]. Interestingly, a recent study showed that KLF5 forms a transcriptional complex with EHF and ELF3 and binds to the promoter region of RAD51 to enhance its transcription, strengthening the homologous recombination repair pathway and consequently inducing chemoresistance in ovarian cancer cells [37]. And in GBM cells, it was identified that aberrantly high activation of a FoxM1-RAD51 transcriptional axis attenuates responsiveness of GBM cells to TMZ treatment [38]. Given that the more molecular mechanisms of RAD51 regulation in different types of cancer are revealed, the more context-dependent mechanisms should be explored to further improve the efficacy of DNA damage agent-induced cell death in the specific cancer treatment. In this study, we showed that miR-124 regulates DNA repair and increases chemosensitivity in vitro and in vivo by suppressing the expression of RAD51. More importantly, given that miR-124 is enriched in the central neuron system and the multifaceted roles of miR-124 have been reported, the novel miR-124-RAD51 axis might be a unique mechanism in GBM cells.

MicroRNA-124 is enriched in the central neuron system and plays a multifaced role in maintaining central neuron system homeostasis and inducing neural differentiation [19, 20]. In glioma, it was reported that loss of brain-enriched miR-124 enhances stem-like traits and invasiveness by targeting SNAI2 [21]. In the field of therapy resistance, it was reported that miR-124 sensitizes GBM cells to TMZ treatment by targeting oncogenic Ras family members, EZH2 and AURKA [23, 29, 38]. Meanwhile, CDK4 and CDK6 were identified as the target of miR-124 and sensitizes brain tumor to radiotherapy [39, 40]. And in the field of immunotherapy, a previous study found that miR-124 inhibits STAT3 signaling to enhance T cell-mediated immune clearance in glioma [26]. Utilization of miR-124 combined with PD-1 antibody might be a good strategy to treat glioblastoma. However, the function of these downstream genes in the field of chemoresistance is mainly on cell proliferation, and it can only partially, and imperfectly explain the mechanism of miR-124 on the TMZ responsiveness. Given that stimulated high activation of DNA repair pathway is involved in TMZ resistance, we hypothesized that miR-124 might govern DNA repair activation. Accordingly, using comet assay and γH2AX staining, we demonstrated that miR-124 overexpression sensitizes GBM cells to TMZ treatment by blocking DNA damage repair. And in the mechanistic study, using bioinformatics analysis, reporter assay, correlation analysis in clinical sample and loss or gain of functional experiments, we showed that RAD51 is a novel and bona fide target of miR-124 in GBM cells. More importantly, the RAD51 rescue experiment further confirms the essential role of miR-124-involved high responsiveness to TMZ in vitro and in vivo.

Although we tried our best to establish a preclinical model to demonstrate the potential effect of combinational utilization of miR-124 expression and TMZ in vivo, we pretreated GBM cells with miR-124 and then established an intracranial orthotopic GBM model. This process did not adequately reflect the therapeutic intervention in the real world. Due to the BBB, it’s hard to deliver the therapeutic reagents to the CNS, especially small nucleic acids. In recent years, delivery tools of materials science help bring different types of therapeutic drug cross the BBB. Actually, lipid nanoparticles were used to encapsulate and protect the functional miRNA from degradation and provide enhanced delivery into the immune cell compartment in glioma [41]. Meanwhile, polymeric nanoparticles were applicable to co-deliver miR-124 with miR-21 antagomiR into the brain to inhibit GBM [42]. More importantly, it was reported to deliver multiple tumor-specific polycistronic miRNAs into glioblastoma by using engineered exosomes [22]. Taken together, all these delivery platforms could cross the BBB companied with therapeutic miRNAs. However, given the complex synthetic and purification process and the expensive costing of above delivery tools, it is still a long way to apply into clinic. Importantly, Jens Niewoehner et al. established a brain shuttle module by manipulating the binding mode of an antibody fragment to the transferrin receptor [43]. This therapeutic protein combined with the antibody fragment targeting transferrin receptor could be a novel strategy to improve survival in GBM patients [44, 45]. Physiological protein transcytosis to the brain, especially transferrin, provides a safer and more efficient method to delivery drug [46]. Therefore, in the future study, transferrin carried small nucleic acids like miR-124 mimics, targeting the transferrin receptor on BBB might be a potential strategy to treat glioblastoma. And anyway, our findings provide a strong rationale for combining TMZ with miR-124 to treat GBM patients and warrant further studies.

## Materials and methods

### Public data and bioinformatic analysis

The miRNA expression data (GSE90603, GSE138764, GSE63319) based on none-coding RNA profiling array in GBM were obtained from the open-access Gene Expression Omnibus (GEO) database [47–49], including microRNA sequencing data from both GBM and adjacent tissues. The mRNA expression data based on mRNA microarray or mRNA sequencing and clinical information in GBM were downloaded from The Cancer Genome Atlas (TCGA) and CGGA databases [50, 51]. miRcode, TarBase, miRDB, miRTarBase were used to predict target genes of miR-124 [52–55]. The differential expression genes analysis, clinical survival analysis and correlation analysis were respectively processed by “limma” package, “survival” package or “psych” package of R studio. All the Venn plots, butterfly plot and correlation scatter plot were painted by “ggplot” package of R project.

### Cell culture and transfection

Human glioblastoma cell lines (U87, U251, A172) and human embryonic kidney cell lines (HEK 293T) were obtained from the American Type Culture Collection (ATCC). All cell lines were routinely authenticated by STR and tested negative for mycoplasma. U-87 MG, U251, A172 and HEK 293T cells were cultivated in Dulbecco’s modified Eagle’s medium (DMEM, Gibco) supplemented with 10% fetal bovine serum. All the cells were incubated with a humidified atmosphere of 5% CO_2_ at 37 °C.

The mimics of miR-124, negative control, miR-124 inhibitor and microRNA inhibitor negative control were obtained from the GenePharma (Shanghai, China). Cells were seeded in 6-well-plates at 50% density before transfection. Using Lipofectamine 2000 (Invitrogen) delivered 20 μM miR-124 mimics or miR-124 inhibitor to cell interior. Total RNA and protein were extracted 48 h after transfection.

### Luciferase report assay

To allow perform the luciferase reporter assay, the PGL3-enhancer-RAD51 3′UTR plasmid was established. The 3′UTR of RAD51 containing the predicted binding site of miR-124 was amplified from whole genome by polymerase chain reaction. The primers used in PCR were showed in Supplementary Table 2. Then the clone fragments were inserted into PGL3-enhancer vector. Before transfection, HEK-293T cells were seeded in 48-well plates at 50% confluence. Successively, the cells were co-transfected with 100 ng pGL3-RAD51-3′UTR, 5 ng PRL-TK and 20 μM miR-124 mimics or negative control by using Lipofectamine 2000 reagent. 48 h after transfection, cells were subjected to lysis and luciferase activity were determined as for a dual-luciferase reporter system, according to the manufacture instructions.

### Co-culture cell competition assay

Stable expressing green fluorescent protein (GFP) or red fluorescent protein (mCherry) GBM cell lines (U87-GFP, U87-mCherry, U251-GFP, U251-mCherry) were established. GFP cells were transfected with miR-Ctrl, meanwhile, miR-124 was overexpressed in mCherry cells. After 24 h of transfection, cells were collected, successively seeded in 6-well plates with 1 × 10^5^ GFP cells and 1 × 10^5^ mCherry cells and cocultured. Before treated with Temozolomide (Selleck, S1237), flow cytometry or fluorescence microscopy imaging were used to detect the number of GFP and Cherry cells as initial control group. Treated with TMZ for 72 h, samples were collected for flow cytometry and fluorescence microscopy imaging. The variation of Cherry/GFP cells ratio was calculated and regarded as the sensitivity of cells to TMZ.

$${The}{contr}{ol\; group}\,\left(c\right){ratio\; of}\frac{{Cherry}}{{GFP}}{cells}\,\left({R}_{c}\right)=\frac{{N}_{{Cherry}}\left(c\right)}{{N}_{{GFP}}\left(c\right)}$$

$${The\; miRNA\; group}\,\left(m\right){ratio\; of}\frac{{Cherry}}{{GFP}}{cells}\,\left({R}_{m}\right)=\frac{{N}_{{Cherry}}\left(m\right)}{{N}_{{GFP}}\left(m\right)}$$

$${The\; variation\; of}\frac{{Cherry}}{{GFP}}{cells\; ratio}\,\left({V}_{R}\right)={|R}_{m}(t)-{R}_{c}(t)|$$

### Western blot analysis

Primary β-actin antibody was obtained from Sigma-Aldrich (Merck, A5441). Mouse RAD51 monoclinal antibody (67024-1-lg), rabbit RAD51 polyclonal antibody (14961-1-AP) and rabbit Caspase3/p17/p19 polyclonal antibody (19677-1-AP) were purchased from Poteintech (China, Wuhan). Detailed antibody information was included in Supplementary Table 1. Total proteins were separated from cells by using RIPA buffer (Solarbio, R0010) supplemented with PMSF (Solarbio, P0100). Protein concentration was quantified using BCA kit (Solarbio, PC0020). In brief, 15 μg of whole-cell lysate from each sample was loaded on a 10% polyacrylamide gel for electrophoresis and transferred onto nitrocellulose filter (NC) membranes. Then, the membranes were blocked in 5% non-fat milk in Tris-buffered saline solution (PH 7.4) containing 0.1% Tween-20 and incubated with primary antibody at a concentration of 1:2500 (for β-actin), 1:5000 (for RAD51) or 1:3000 (for Caspase3) overnight at 4 °C. The secondary antibodies were used at a concentration of 1:10,000 for 1 h at room temperature. Enhanced chemiluminescence reagent was used to visualize the protein.

### Single-cell gel electrophoresis (comet) assay

Comet assay was performed as previously described [56]. Proliferating U-87 MG and A172 cells were transfected with miR-Ctrl or miR-124 mimics. After 24 h, transfected cells were treated or not with 500 μM TMZ for 4 h and repaired for 15 h to prepare single cell samples. Briefly, treated and untreated cells were collected and resuspended in ice-cold PBS (without Mg^2+^ and Ca^2+^) at 1 × 10^5^ cells per mL. Combined the cells with low-melt agarose at 1/10 ratio at 37 °C, mixed well by pipetting, and immediately transferred 75 μL suspension onto frosted glass slides. The slides were successively placed in pre-chilled Lysis Buffer and Alkaline Solution (Beyotime, C2041M) at 4 °C in the dark, after the agarose solidified. Slides were then subjected to alkaline electrophoresis (1 V per cm of distance between electrodes) for 20 min in alkaline solutions and stained with propidium iodide. DNA damage was quantified for the average Tail DNA% of 6 views, a fraction of tail DNA intensity with total DNA intensity, analyzed by the OpenComet in ImageJ software.

### Real-time quantitative PCR analysis

Total RNA was isolated from cells using TRIzol reagent (Thermo Fisher Scientific, 15596018CN). Reverse transcription for mRNA was performed using PrimeScript RT reagent Kit (Perfect Real Time) (Takara, RR047A) according to the manufacturer’s protocol. Mir-X miRNA First-Strand Synthesis Kit (Takara, 638313) for miR-124 reverse transcription was purchased from Takara. β-actin and U6 snRNA (sn-RNU6) were used as normalization controls. All the primer of qPCR were acquired from Tsingke (Supplementary Table 2). Bio-Rad C1000 Thermal Cycler (Bio-Rad, California, CA, USA) was used to detect the relative expression of mRNA or microRNA and data were analyzed by using the 2^-(ΔΔCt) method.

### Immunofluorescence and confocal microscopy imaging

Cells transfected with miR-Ctrl or miR-124 were plated on coverslips and treated with 200 μM TMZ for 24 h. After treatment, 4% paraformaldehyde were used to fix cells for 15 min. Before primary antibody incubation, cells were blocked with 3% bovine serum albumin containing 0.3% Triton-x-100 for 1 h at room temperature. Then cells were incubated with primary antibody against RAD51 (Abcam, ab133534, 1:1000) or γ-H2AX (ABclonal, AP0099, 1:300) in 4 °C overnight. Coralite594-conjugated secondary antibody (Proteintech, SA00013-4, 1:500) or coralite488-conjugated secondary antibody (Proteintech, SA00013-2, 1:1000) was used to combine primary antibody for 1 h. Nuclei were counterstained with 4′,6-diamidino-2-phenylindole (DAPI). Phase images were captured by confocal laser scanning microscope FV3000 series (Olympus) at a magnification of 100× and processed by ImageJ.

### Cell apoptosis and viability analysis

After transfected with mimics miR-Ctrl or miR-124, cells were treated with 200 μM TMZ for 24 h. Cells were then collected and resuspended in PBS. FITC Annexin-V Apoptosis Detection Kit I (BD Bioscience, 556547) was used to stain the valgus phosphatidylserine and the FITC positive cells were analyzed by flow cytometry. In xenograft orthotopic tumor tissues, apoptosis of tumor cells was detected by TUNEL staining using an In Situ Cell Death Detection Kit, Fluorescein (Servicebio, G1504) according to the manufacturer’s protocol. The nuclei were then counterstained with DAPI. Phase images were captured by confocal laser scanning microscope FV3000 series (Olympus) at a magnification of 100× and processed by ImageJ.

Cells were transfected with mimics or microRNA inhibitor and then collected to seed at 3.0 × 10^3^ cells per well onto 96-well plates. Cells were treated with TMZ and counted by Cell Counting Kit-8 assay (CCK-8) (Yeasen, 40203ES80) at 0, 24, 48 h. Cell survival was calculated by normalizing the absorbance to that of untreated controls.

### Animal orthotopic model

Five-week-old male BALB/c nude mice were purchased from the GemPharmatech LLC (Chengdu, China). All animal procedures were approved by the Laboratory Animal Welfare and Ethics Committee of Air Force Medical University (AFMU). To establish an intracranial tumor model, U87 (5 × 10^5^ in 5 μL PBS) cells with stable expression miR-Ctrl or miR-124, were stereo-tactically implanted into the nude mouse brain by 69100 Rotational Digital Stereotaxic Frame (RWD Life Science). During the surgery, a Hamilton syringe was inserted at lateral 2 mm and ventral 1 mm to the bregma to a depth of 3.5 mm and then pulled back 0.5 mm to allow space for tumor cells. Injection process was sustained 10 min at a speed of 0.5 μL/min and the Hamilton syringe was held for 10 min to avoid the tumor cells overflowed before syringe extraction [57]. After 5 days of intracranial injection, nude mice were treated with normal saline or TMZ (20 mg/kg) by intraperitoneal injection for 4 weeks (once every other day) [58]. The number of surviving nude mice was used to plot Kaplan–Meier survival curve. The brains of nude mice were obtained and embedded with Optimal cutting temperature compound (OCT) for further analysis.

### Statistical analysis

Each experiment was repeated at least three times and data analyses were performed using GraphPad Prism version 8.0.2 (GraphPad, San Diego, USA) and R project (version 4.3.0). All data were expressed as mean values ± SD. The student *t*-test, one-way analysis of variance (ANOVA), log-rank text and Person correlation test were used to determine significant differences between the groups with *p* values of less than 0.05 considered statistically significant.

## Supplementary information

supplementary material figure and legend

supplementary material original WB

## Data Availability

The data supporting the finding of this study are available from the corresponding authors upon reasonable request.
