# Supplementary material for: Overexpression of miR-124 enhances the therapeutic benefit of TMZ treatment in the orthotopic GBM mice model by inhibition of DNA damage repair
Source: Cell Death Dis. 2025 Jan 26;16(1):47. doi: 10.1038/s41419-025-07363-z (PMC11770086; doi:10.1038/s41419-025-07363-z)
Supplement: Supplementary file 1 — supplementary material figure and legend [file 41419_2025_7363_MOESM1_ESM.docx]

**Supplemental figures:**


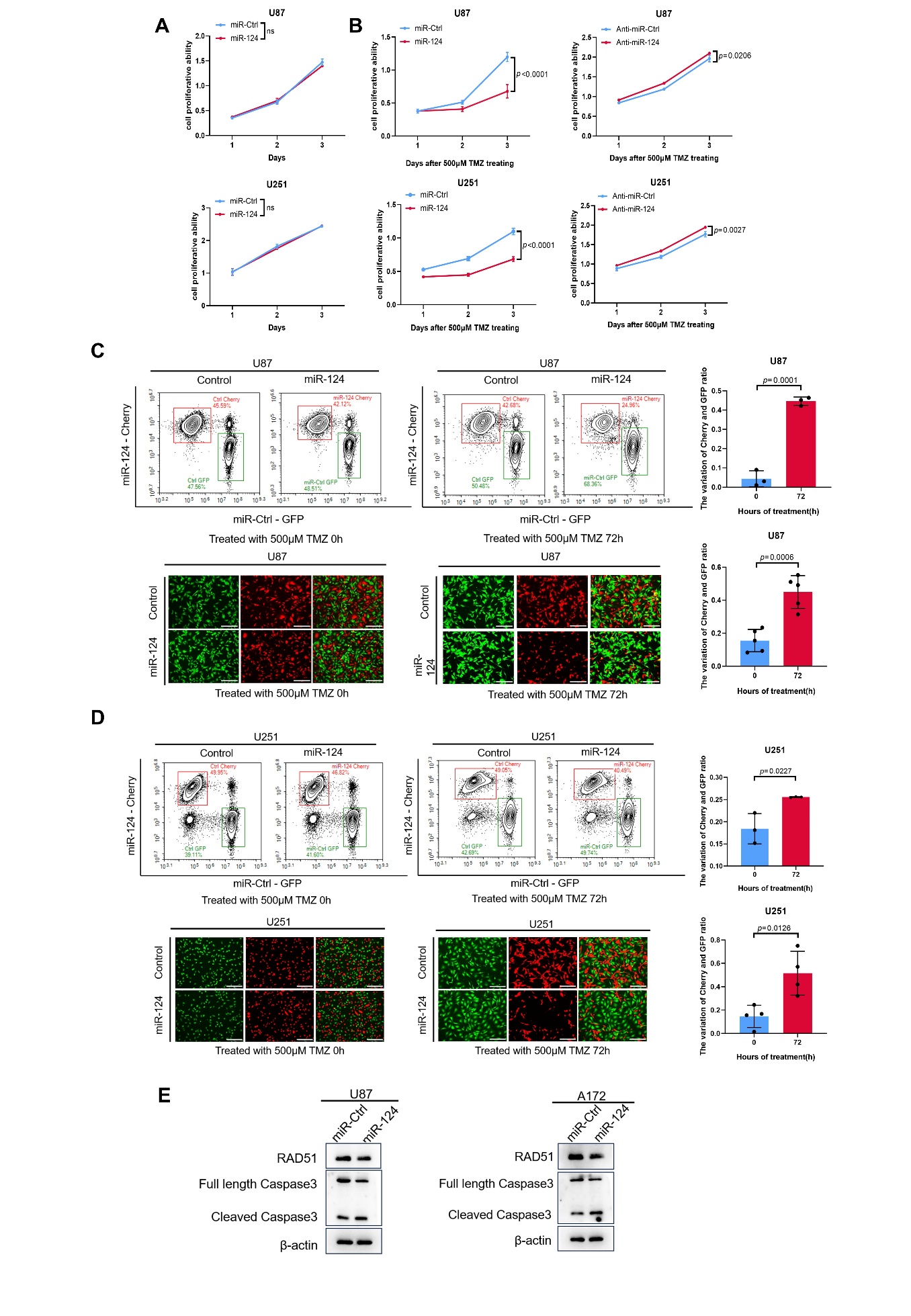


**Supplemental Figure 1.** **MiR-124 enhances the sensitivity of glioma cell lines to TMZ treatment.** (A-B) CCK8 assay was used to assess the cell proliferative ability of different groups. U87 and U251 cells transfected with miR-Ctrl or miR-124 in (A), U87 and U251 cells transfected with miR-Ctrl or miR-124 followed by 500 μM TMZ treatment in (B, left), U87 and U251 cells transfected with anti-miR-Ctrl or anti-miR-124 followed by TMZ treatment in (B, right). (C-D) Representative images of flow cytometry (left, top) and fluorescence (left, bottom) and the variation ratio of Cherry / GFP cells (right) from coculture fluorescence competition assay in U87 and U251 cells exposed to 500 μM TMZ, with or without miR-124 overexpression. Scale bar, 300 μm. (E) Western blot analysis of cleaved Caspase3 expression in U87 and A172 cells exposed to TMZ (200 μM, 24 h), with or without miR-124 overexpression. Above data are presented as means ± SD. All experiments were repeated at least 3 times. *P* values were calculated using unpaired Student’s t test.


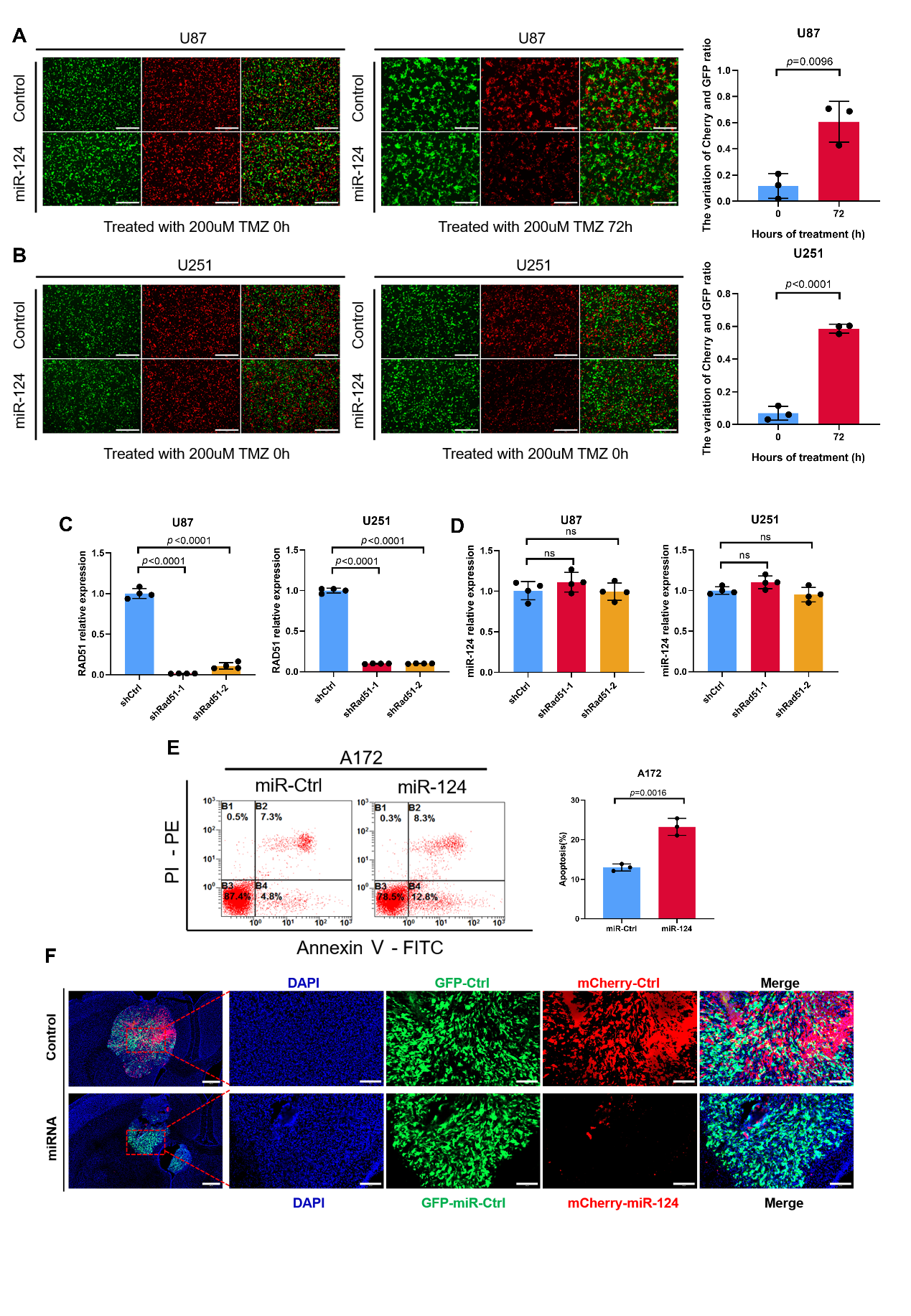


**Supplemental Figure 2.** **MiR-124 sensitizes glioma cell lines to TMZ treatment.** (A-B) Representative lower exposure images of fluorescence imaging (left) and the variation ratio of Cherry/GFP cells (right) from co-culture cell competition assay in U87 and U251 cells exposed to 200 μM TMZ, with or without miR-124 expression. Scale bar, 750 μm. (C-D) qRT-PCR analysis of miR-124 expression in U87 and U251 cells with or without RAD51 depletion. (E) Representative images (left) and apoptosis rate (right) of cell apoptosis assay in A172 cells exposed to TMZ (200 μM, 24h) with or without miR-124 expression. (F) Representative lighter exposure images of co-culture fluorescence cell competition assay in vivo. Above data are presented as means ± SD, n=3 independent experiments. All experiments were repeated at least 3 times. Above data are presented as means ± SD. P values were calculated using unpaired Student’s t test.


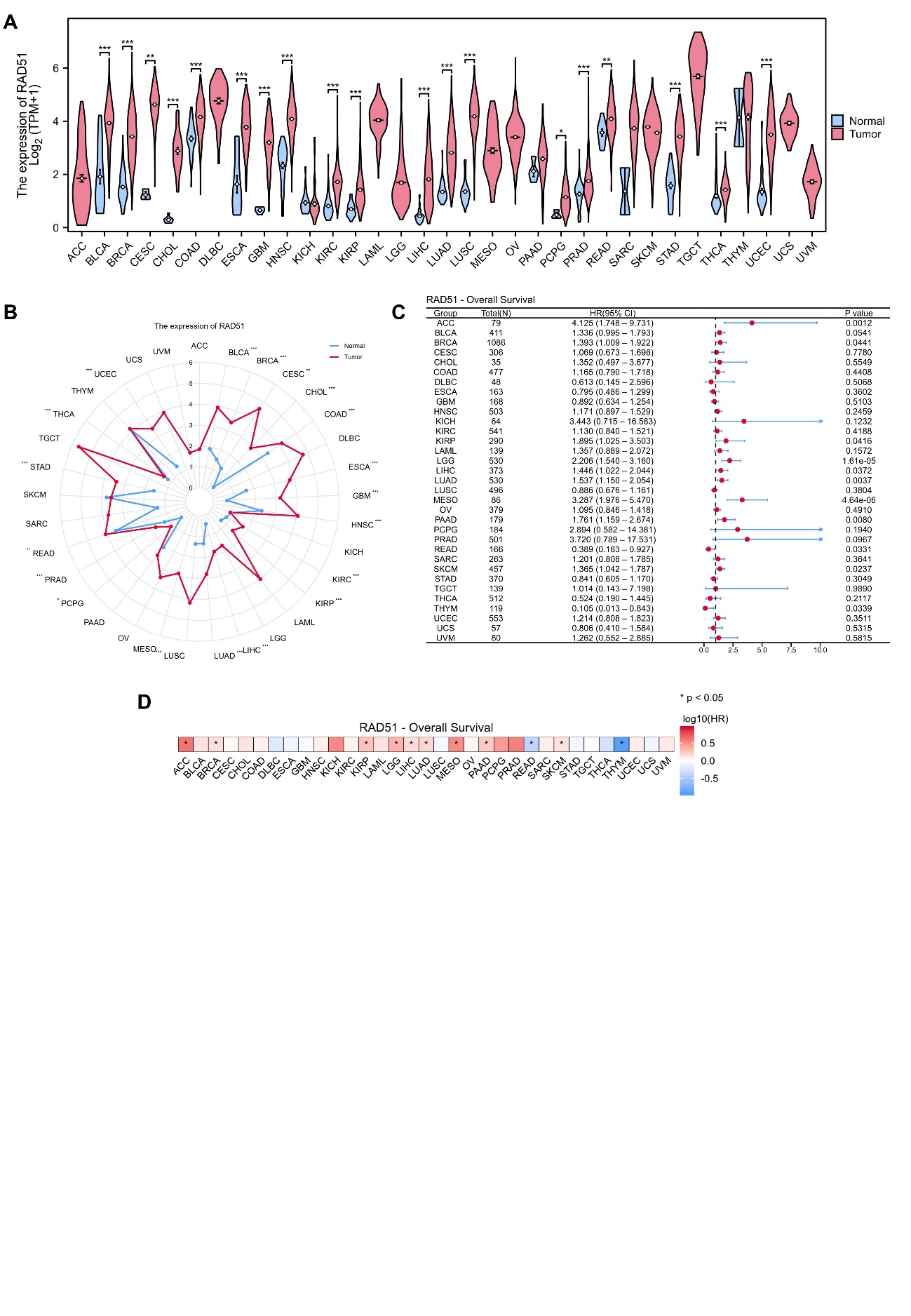


**Supplemental Figure 3.** **RAD51 is upregulated in various cancers and correlated with poor survival.** (A) RAD51 mRNA expression between tumor and peritumor tissues in 33 cancer types in TCGA datasets. (B) Radar chart showed RAD51 mRNA expression between tumor and peritumor tissues in 33 cancer types in TCGA datasets. (C and D) Forest plot (C) and prognostic heat map (D) showing the association of RAD51 expression with the overall survival in 33 cancer types in TCGA datasets. *P* values in (A) and (B) were calculated using unpaired Student’s t test, and in (C) and (D) were calculated using a log-rank test.


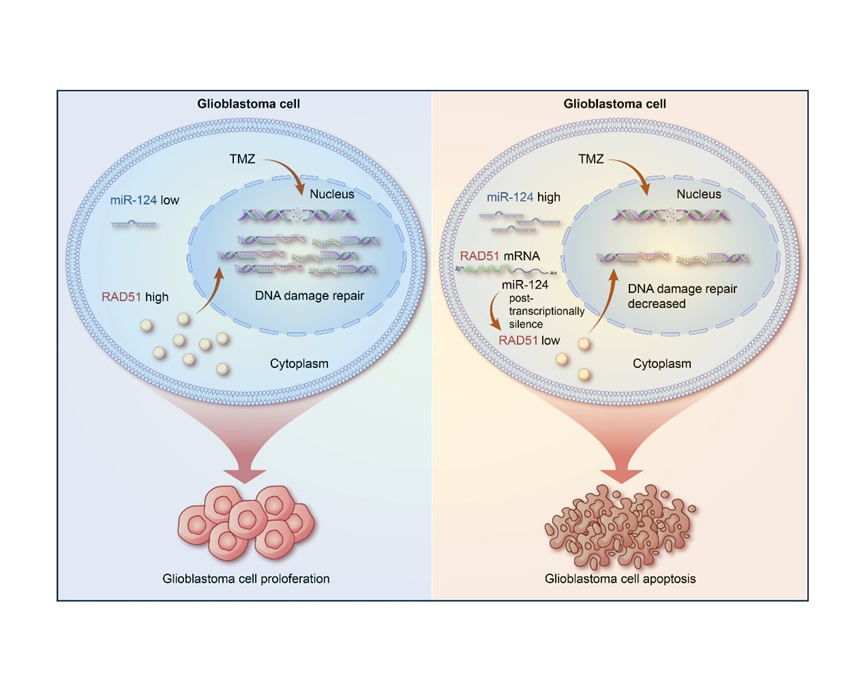


**Supplemental Figure 4.** **Working model of miR-124-RAD51 signaling in chemosensitivity in GBM.**

**Supplemental Table 1. Antibodies used in this paper:**

| **Antibody** | **Company** | **Cat#** |
| --- | --- | --- |
| RAD51 rabbit polyclonal antibody | Proteintech | 14961-1-AP |
| RAD51 monoclonal antibody | Proteintech | 67024-1-lg |
| Caspase 3/p17/p19 polyclonal antibody | Proteintech | 19677-1-AP |
| Coralite594-conjugated goat anti-mouse lgG | Proteintech | SA00013-3 |
| Coralite488-conjugated goat anti-mouse lgG | Proteintech | SA00013-1 |
| Coralite594-conjugated goat anti-rabbit lgG | Proteintech | SA00013-4 |
| Coralite488-conjugated goat anti-rabbit lgG | Proteintech | SA00013-2 |
| Anti-Ki67 mouse mAb | Servicebio | GB121141 |
| Anti-RAD51 antibody | Abcam | ab133534 |
| Phospho-histone H2AX-S139 rabbit pAb | Abclonal | ApP0099 |

**Supplemental Table 2. Sequences of primers and oligo mimics:**

| **Primers for PCR** |  | **sequence 5'-3'** |
| --- | --- | --- |
| miR-124 | Forward Primer | TTGATATCGATGCTGTGGTCCCTTCCTCC |
|  | Reverse Primer | GGGTTTAAACCCACCCCACGGGCTCCG |
| RAD51 ORF | Forward Primer | GCTCTAGAATGGATTACAAGGACGACGATGACAAGGCAATGCAGATGCAGCTT |
|  | Reverse Primer | CGCGGATCCTCAGGAAGACAGGGAGAGTCGT |
| RAD51 3'UTR | Forward Primer | GGGGTACCCTCTACAGGCCTCTTCCTGT |
|  | Reverse Primer | CCCTCGAGCAGACAACTTAAAAACCTGCTTGAG |
| **Primers for qPCR** |  | **sequence 5'-3'** |
| β-Actin | Forward Primer | CATGTACGTTGCTATCCAGGC |
|  | Reverse Primer | CTCCTTAATGTCACGCACGAT |
| RAD51 | Forward Primer | CGAGCGTTCAACACAGACCA |
|  | Reverse Primer | GTGGCACTGTCTACAATAAGCA |
| U6 | Forward Primer | GTGCTCGCTTCGGCAGCACATATAC |
|  | Reverse Primer | AAAAATATGGAACGCTTCACGAATTTG |
| miR-124 | Forward Primer | TAAGGCACGCGGTGAATGCCAA |
|  | Reverse Primer | Universal Primer (QIAGEN) |
| **Oligo mimics** |  | **sequence 5'-3'** |
| has-miR-124 | Sense | UAAGGCACGCGGUGAAUGCCAA |
|  | Antisense | GGCAUUCACCGCGUGCCUUAUU |
| Negative control | Sense | UUCUCCGAACGUGUCACGUTT |
|  | Antisense | ACGUGACACGUUCGGAGAATT |
| has-miR-124 inhibitor | Sense | UUGGCAUUCACCGCGUGCCUUA |
