# Supplementary material for: Overexpression of miR-124 enhances the therapeutic benefit of TMZ treatment in the orthotopic GBM mice model by inhibition of DNA damage repair
Source: Cell Death Dis. 2025 Jan 26;16(1):47. doi: 10.1038/s41419-025-07363-z (PMC11770086; doi:10.1038/s41419-025-07363-z)

**Figure 4E**

U251 cell lines:

Grouped into: miR-Ctrl, miR-124

β-actin (45 KD) RAD51(37 KD)


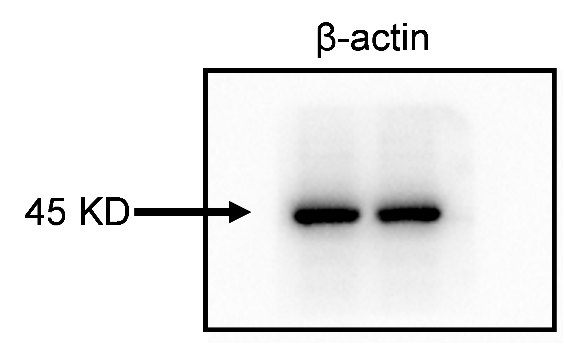

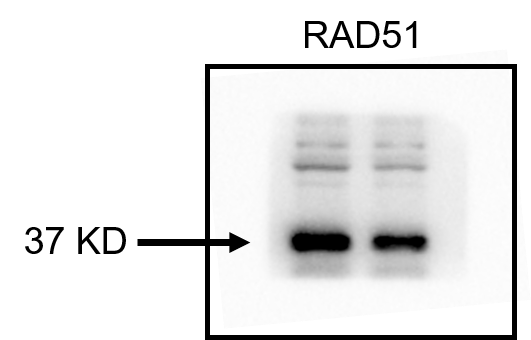


U87 cell lines:

Grouped into: miR-Ctrl, miR-124

β-actin (45 KD) RAD51(37 KD)


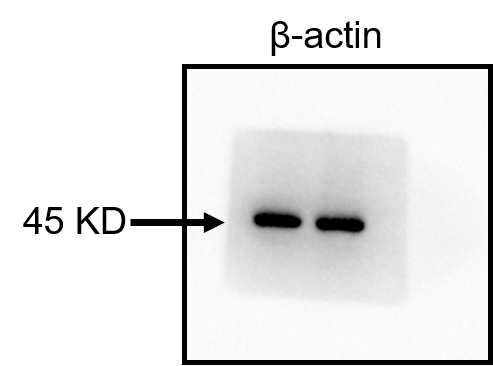

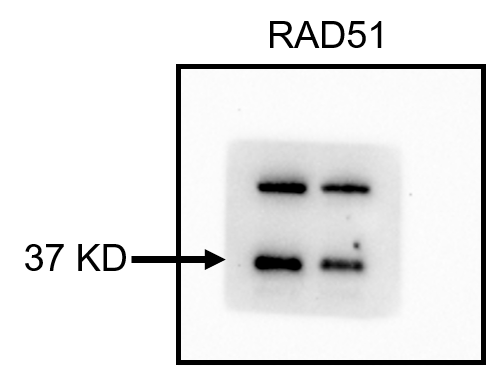


A172 cell lines:

Grouped into: miR-Ctrl, miR-124

β-actin (45 KD) RAD51(37 KD)


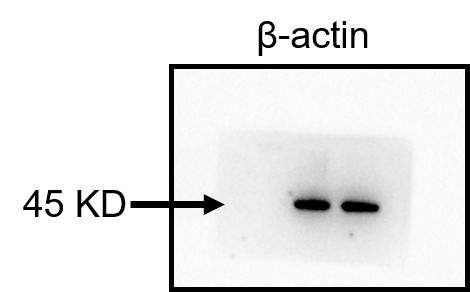

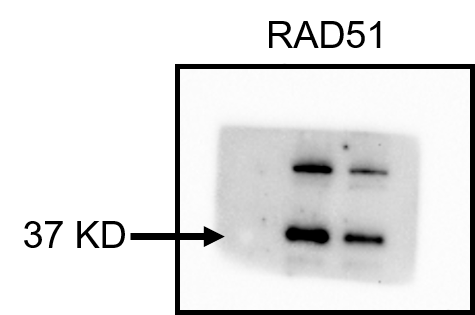


**Figure 4G**

U251 cell lines:

Grouped into: Anti-miR-Ctrl, Anti-miR-124

β-actin (45 KD) RAD51(37 KD)


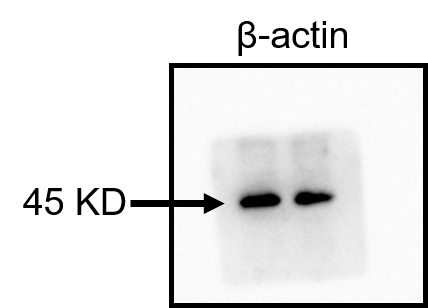

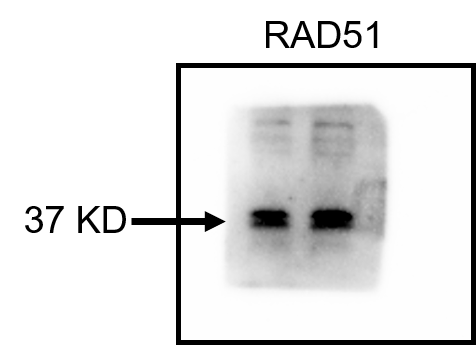


U87 cell lines:

Grouped into: Anti-miR-Ctrl, Anti-miR-124

β-actin (45 KD) RAD51(37 KD)


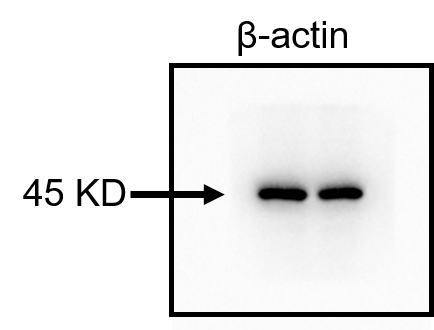

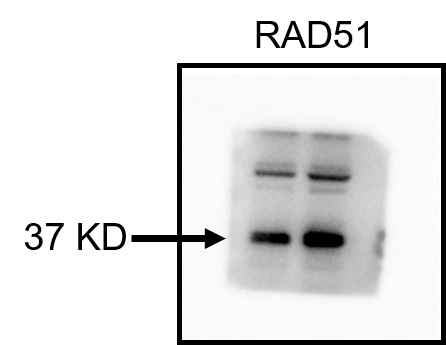


A172 cell lines:

Grouped into: Anti-miR-Ctrl, Anti-miR-124

β-actin (45 KD) RAD51(37 KD)


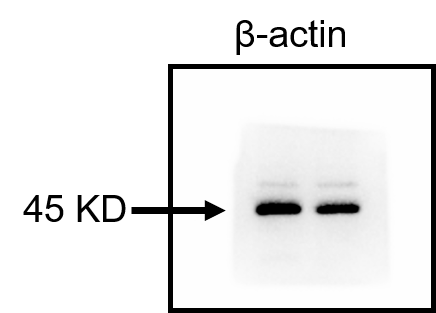

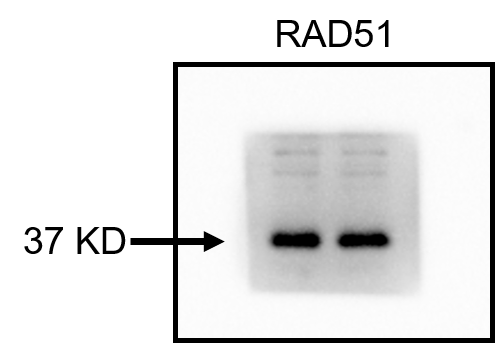


**Figure 5C and D**

U87 cell lines:

Grouped into: shCtrl, shRAD51-1, shRAD51-2

β-actin (45 KD) RAD51(37 KD)


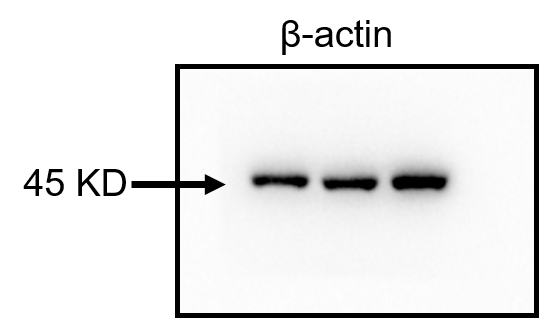

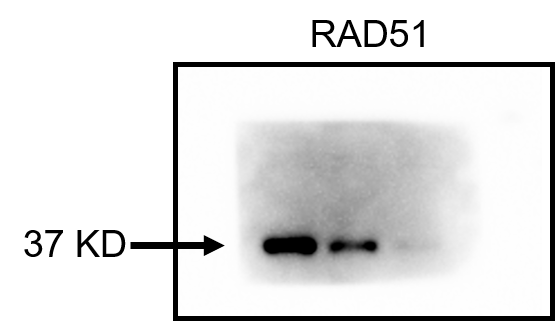


U251 cell lines:

Grouped into: shCtrl, shRAD51-1, shRAD51-2

β-actin (45 KD) RAD51(37 KD)


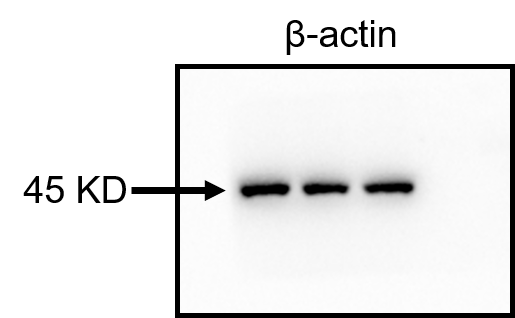

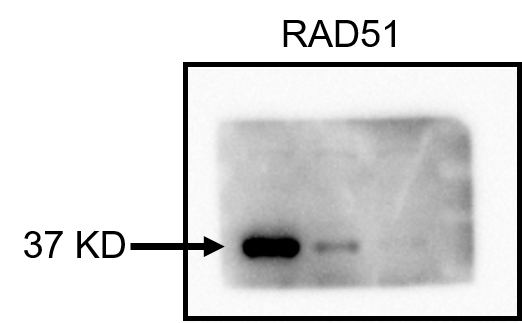


**Figure 6A and B**

U87 cell lines:

Grouped into: miR-Ctrl plus Vector, miR-124 plus Vector, miR-Ctrl plus RAD51, miR-124 plus RAD51

β-actin (45 KD) RAD51(37 KD)


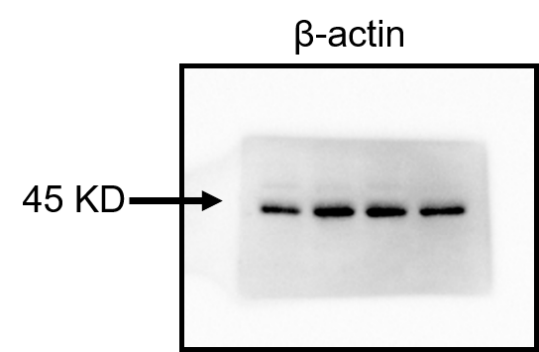

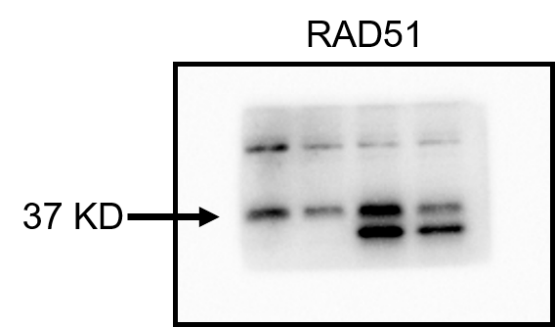


U251 cell lines:

Grouped into: miR-Ctrl plus Vector, miR-124 plus Vector, miR-Ctrl plus RAD51, miR-124 plus RAD51

β-actin (45 KD) RAD51(37 KD)


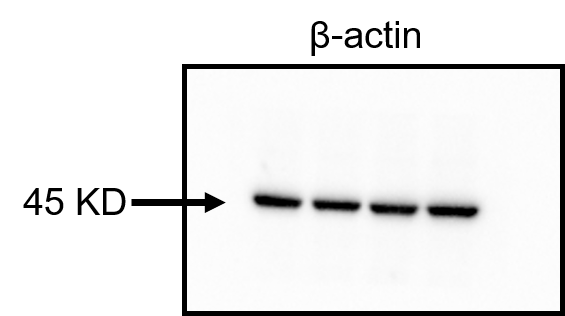

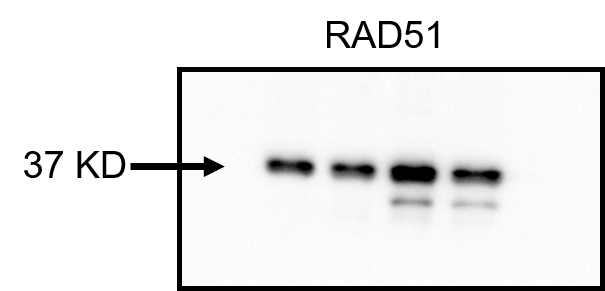


**Supplementary Figure 1E**

U87 cell lines:

Grouped into: miR-Ctrl, miR-124

β-actin (45 KD) RAD51(37 KD)


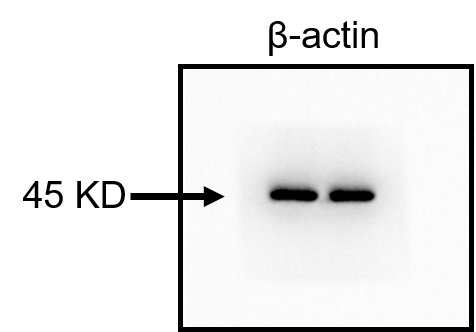

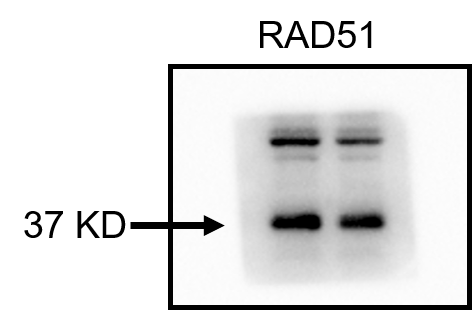


Full length Caspase3 (32 KD) Cleaved Caspase3 (17 KD)


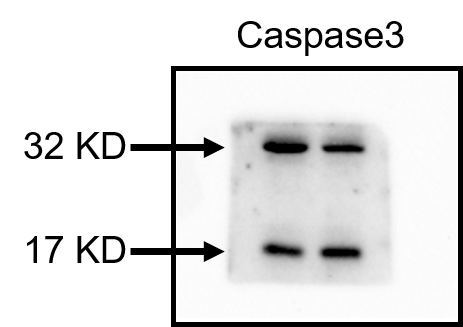


A172 cell lines:

Grouped into: miR-Ctrl, miR-124

β-actin (45 KD) RAD51(37 KD)


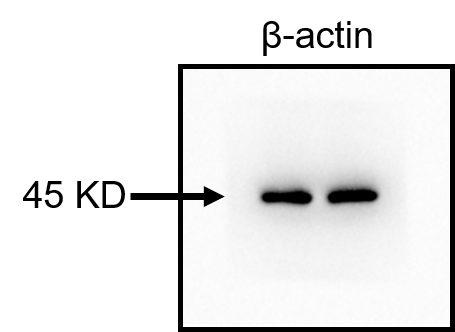

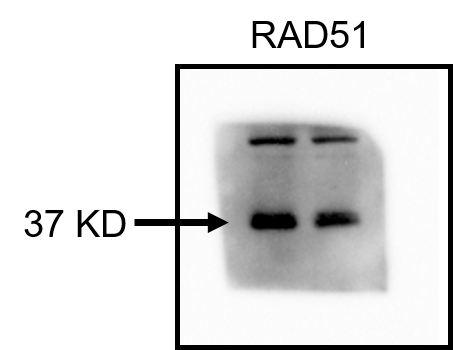


Full length Caspase3 (32 KD) Cleaved Caspase3 (17 KD)


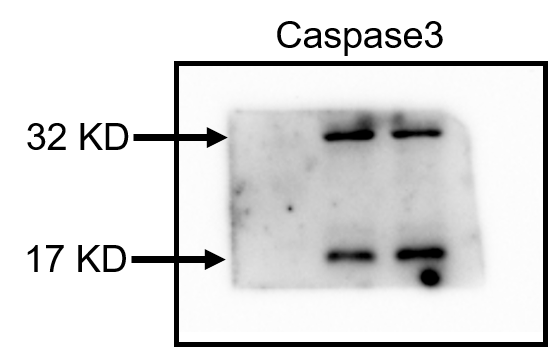

Supplement: Supplementary file 2 — supplementary material original WB [file 41419_2025_7363_MOESM2_ESM.docx]
